# The evolutionary history of polycotylid plesiosaurians

**DOI:** 10.1098/rsos.172177

**Published:** 2018-03-28

**Authors:** V. Fischer, R. B. J. Benson, P. S. Druckenmiller, H. F. Ketchum, N. Bardet

**Affiliations:** 1 Department of Geology, Université de Liège, 14 allée du 6 Août, Liège 4000, Belgium; 2 Department of Earth Sciences, University of Oxford, South Parks Road, Oxford OX1 3AN, UK; 3 University of Alaska Museum and Department of Geosciences, University of Alaska Fairbanks, 1962 Yukon Drive, Fairbanks, AK 99775, USA; 4 Oxford University Museum of Natural History, Parks Road, Oxford OX1 3PW, UK; 5 CR2P CNRS-MNHN-UPMC Paris 6, Département Origines et Evolution, Muséum National d'Histoire Naturelle, CP 38, 57 rue Cuvier, 75005 Paris, France

**Keywords:** evolutionary radiation, biotic turnover, Sauropterygia, Plesiosauria, morphology

## Abstract

Polycotylidae is a clade of plesiosaurians that appeared during the Early Cretaceous and became speciose and abundant early in the Late Cretaceous. However, this radiation is poorly understood. *Thililua longicollis* from the Middle Turonian of Morocco is an enigmatic taxon possessing an atypically long neck and, as originally reported, a series of unusual cranial features that cause unstable phylogenetic relationships for polycotylids. We reinterpret the holotype specimen of *Thililua longicollis* and clarify its cranial anatomy. *Thililua longicollis* possesses an extensive, foramina-bearing jugal, a premaxilla–parietal contact and carinated teeth. Phylogenetic analyses of a new cladistic dataset based on first-hand observation of most polycotylids recover *Thililua* and *Mauriciosaurus* as successive lineages at the base of the earliest Late Cretaceous polycotyline radiation. A new dataset summarizing the Bauplan of polycotylids reveals that their radiation produced an early burst of disparity during the Cenomanian–Turonian interval, with marked plasticity in relative neck length, but this did not arise as an ecological release following the extinction of ichthyosaurs and pliosaurids. This disparity vanished during and after the Turonian, which is consistent with a model of ‘early experimentation/late constraint’. Two polycotylid clades, Occultonectia clade nov. and Polycotylinae, survived up to the Maastrichtian, but with low diversity.

## 1. Introduction

Polycotylidae is a peculiar clade of xenopsarian plesiosaurs whose members typically exhibit short necks and elongated skulls [1–5]. These body proportions resemble those of the pliosaurids, another clade within Plesiosauria, and many historical schemes classified these clades together (often as ‘pliosauroids’). However, the cladistic analysis of O'Keefe [6] and many subsequent works (e.g. [3,7–9]; but see Smith & Dyke [10] and Druckenmiller & Russell [11]) recovered polycotylids as a derived clade of plesiosauroids (generally long-necked plesiosaurians), that secondarily evolved short necks, independently of pliosaurids. Polycotylids therefore provide a striking example of convergent evolution in marine reptiles.

**Figure 1.**
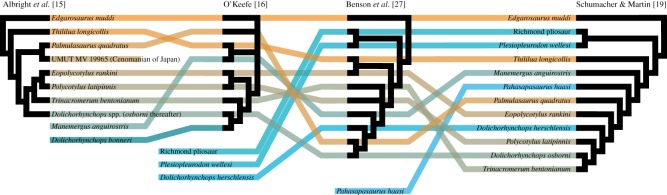
Evolution and instability of the phylogenetic relationships among polycotylid plesiosaurians, using the previous main works on this topic. The colour coding matches the order of branching in the phylogeny of Albright *et al*. [15]; taxa subsequently added are coloured in blue hues.

The earliest polycotylids occur possibly during the Aptian [12] and certainly by the Albian [13,14]. The clade then rose in diversity during the early Late Cretaceous. However, phylogenetic uncertainties obscure their evolutionary history and the shape of their radiation. Indeed, the phylogenetic relationships of early polycotylids have rarely been evaluated by focused analysis and have proved controversial (see figure 1 for a summary of the main previous phylogenetic hypotheses of polycotylid plesiosaurians). Albright *et al*. [15] recovered *Edgarosaurus muddi* from the Albian of North America and *Thililua longicollis* from the Turonian of Morocco as the earliest-diverging polycotylids, forming successive sister groups to the clade uniting the two subfamilies Palmulasaurinae and Polycotylinae. Of these, Polycotylinae includes the youngest polycotylids, *Dolichorhynchops* spp., *Trinacromerum bentonianum*, *Polycotylus latipinnis* and *Eopolycotylus rankini*, of Turonian–Campanian age. O'Keefe [16] modified the matrix of Albright *et al*. [15] and found less resolution of relationships within Polycotylidae, with hypothesized members of Palmulasaurinae acting as wildcard taxa. O'Keefe [16] nevertheless recovered a monophyletic Polycotylinae and proposed a sister taxon relationship between the Moroccan Turonian taxa *Thililua longicollis* and *Manemergus anguirostris*, occurring outside of Polycotylinae.

Most other phylogenetic analyses of Plesiosauria have been wider in scope, but included a smaller sample of polycotylids effectively as placeholders for the clade [6,8,10,11,17], or included larger numbers of polycotylids, but deviated substantially from the topologies of Albright *et al*. [15] and O'Keefe [16]: Ketchum & Benson [7] and Benson & Druckenmiller [3]. Otero [18] incorporated a large number of polycotylids, inadvertedly using preliminary scores of R.B.J. Benson. Most recently, Schumacher & Martin [19] and Fischer *et al*. [9] included a relatively large sample of polycotylids and recovered topologies that are broadly similar to that of Albright *et al*. [15] (figure 1).

Comparison of the large set of previous analyses highlights the uncertain phylogenetic placement of the Turonian, Moroccan polycotylids *Thililua longicollis* and *Manemergus anguirostris* (figure 1). *Thililua longicollis* is an enigmatic taxon that so far was only described in brief [20] and deviates from the general body proportions of other polycotylids in having a long neck, composed of 30 elongated cervical vertebrae bearing lateral ridges, similar to those of long-necked plesiosaurs from several other clades (Microcleididae, Cryptoclididae and Elasmosauridae). This taxon may provide critical information on the early diversification of polycotylids, because its phylogenetic position has varied intensely since its creation and because of its highly unusual anatomy. Indeed, *Thililua longicollis* has been described by Bardet *et al*. [20] as a polycotylid with a bizarre cranial architecture including a strongly reduced jugal and a strongly reduced, nearly absent postorbital, in addition to features previously unknown among polycotylids such as an elongated neck and reniform orbits otherwise present in elasmosaurids (e.g. [21–23]), and in fossil and recent species of *Gavialis*, an extant crocodilian (e.g. [24]). *Manemergus anguirostris* is another polycotylid from the same age and locality [25] that shares several features within *Thililua longicollis*, raising concerns about its taxonomic validity. Although O'Keefe [16] found *Thililua longicollis* and *Manemergus* as sister taxa, both Ketchum & Benson [7] and Druckenmiller & Knutsen [26] recovered *Manemergus* as a derived polycotylid while *Thililua longicollis* was found outside of Polycotylidae, being closely related with the leptocleidid *Nichollssaura borealis*. Benson *et al*. [27] recovered *Thililua longicollis* as a relatively early diverging polycotylid and *Manemergus* as a derived polycotylid. Schumacher & Martin [19] recovered both *Thililua longicollis* and *Manemergus anguirostris* as members of a basal grade outside of Palmulasaurinae and Polycotylinae. A similar position was found by Fischer *et al*. [9] for *Thililua longicollis*.

*Plesiopleurodon wellesi*, from the Cenomanian of Wyoming (USA; [28]), also embodies the uncertainties of early polycotylid evolution and relationships. Indeed, this taxon was regarded as a pliosaurid/‘pliosauroid’ for decades [28,29] before being recognized as a polycotylid [3,7,19,27]. Moreover, the phylogenetic relationships of recently described Middle Cretaceous polycotylids *Eopolycotylus rankini* from the late Early Turonian of the USA [15], *Dolichorhynchops tropicensis* from the Early Turonian of USA [30], *Sulcusuchus erraini* from the Late Campanian–Early Maastrichtian of Argentina [31,32] and *Mauriciosaurus fernandezi* from the Early Turonian of Mexico [33] have never or rarely been tested [19].

Because of this lack of consensus about their phylogenetic relationships and incomplete inclusion of certain early Late Cretaceous species from current phylogenies, the dynamics of the evolutionary radiation of polycotylids are currently unknown. Here, we (i) revise the morphology and taxonomy of a key taxon to understand the Late Cretaceous radiation of polycotylids, *Thililua longicollis* from the Turonian of Morocco, (ii) analyse the phylogenetic relationships of all polycotylids using the largest phylogenetic dataset ever devoted to Plesiosauria, including first-hand observations on most polycotylids, and (iii) investigate their pattern of morphospace occupation over time using an updated dataset summarizing the Bauplan of polycotylids.

The radiation of polycotylids peaked during the earliest Late Cretaceous and coincided with a burst of disparity, producing markedly distinct lineages during the Cenomanian–Turonian. *Thililua longicollis* and *Mauriciosaurus* are recovered as the successive sister taxa of Polycotylinae, suggesting that wide variations of body shape took place prior to the stabilization of the streamlined, generally short-necked, and morphologically constrained polycotyline morphotype. Two phylogenetically distant clades of polycotylids survived to at least the Late Campanian, although severely diminished in diversity and disparity.

## 2. Material and methods

### 2.1. Institutional abbreviations

MHNGr: Muséum d'Histoire Naturelle de Grenoble, Grenoble, France. MNA: Museum of Northern Arizona, Flagstaff, USA. QM: Queensland Museum, Brisbane, Australia. ROM: Royal Ontario Museum, Toronto, Canada. RSM: Royal Saskatchewan Museum, Regina, Canada. SMNK: Staatliches Museum für Naturkunde Karlsruhe, Karlsruhe, Germany.

### 2.2. Phylogenetic analysis

We used the dataset assembled by Benson & Druckenmiller [3], incorporating subsequent modifications on short-necked plesiosaurs [9,34] and elasmosaurids [35]. This resulted in a matrix containing 118 taxa and 270 characters, representing the most complete phylogenetic data matrix of Plesiosauria to date. We modified the scores of *Thililua longicollis* based on new personal observations of the type material by V.F., and we added the following taxa: *Eopolycotylus rankini* (Albright *et al*. [15]), *Manemergus anguirostris* (R.B.J.B. 2010, personal observations on the holotype SMNK-PAL 3861; [25]), *Dolichorhynchops tropicensis* [30], *Georgiasaurus penzensis* [36], *Dolichorhynchops* sp. (R.B.J.B. & P.S.D. 2011, personal observations on ROM 29010; [37]), *Dolichorhynchops herschelensis* (P.S.D. 2006, personal observations on RSM P2310.1; [38]), *Sulcusuchus erraini* [31] and *Mauriciosaurus fernandezi* [33]. We conducted a maximum-parsimony analysis in TNT v. 1.5 [39], using the parsimony ratchet. We extended the RAM allocation to 1000 megabytes (mxram 1000;) and the memory to 50 000 trees (hold 50 000;). We employed the following options for the parsimony ratchet (‘New technology search’ in TNT v. 1.5 [39]): ratchet and drift activated, 100 ratchet iterations; all other options are those by default. The trees obtained from the parsimony ratchet were then used as a basis for a classic tree bisection reconnection (TBR) search (‘trees from RAM’ option in ‘Traditional search’ in TNT v. 1.5 [39]). We used an *a posteriori* approach to detect and remove wildcard taxa developed by Pol & Escapa [40] and directly implemented in TNT v. 1.5 [39]. This pruned dataset was then reanalysed using the exact same procedures described above (see also electronic supplementary material, ESM1, ESM2 (full phylogenetic dataset), ESM3 (taxon ages for the full phylogenetic dataset), ESM4 (pruned phylogenetic dataset), ESM6 (taxon ages for the pruned phylogenetic dataset) and ESM8 (R script)). Strict consensus trees, throughout, were constructed using the R package ape v. 4.1 [41] and so do not employ the collapsing rules of TNT. Bremer decay and resampling methods to estimate the robustness of nodes (standard bootstrapping and jackknifing, 1000 iterations) were computed in TNT v. 1.5 [39] for both datasets.

### 2.3. Phylogenetic diversity

We computed the evolution of the phylogenetic diversity of polycotylids, using all the most parsimonious trees arising from the analysis of the full and pruned datasets. All tips were dropped with the exception of polycotylids and *Leptocleidus capensis*, in order to have information about the branch leading to Polycotylidae during the earliest Cretaceous. *Leptocleidus capensis* was chosen because it is the oldest unambiguous member of Leptocleididae, the sister clade of Polycotylidae. Trees were time scaled using both the ‘equal’ and ‘basic’ methods of branch length reconstruction (e.g. [42]), using the ape v. 4.1 [41] and paleotree v. 2.7 packages [43] in R v. 3.4.1 [44]. We computed the median phylogenetic diversity and 95% confidence intervals using the same packages, based on the trees from the full and pruned datasets, resulting in four distinct curves. Caution should be exercised in interpreting the declining phase of diversity as this approach extrapolates ghost lineages back in time only [45]. This can give rise to Signor–Lipps-like edge effects [46] at the end of the evolutionary history of a clade, resulting in the appearance of a gradual decline of phyletic diversity even if extinction was abrupt [47]. Furthermore, mis-specification of the phylogeny can result in the erroneous inference of high early diversity, i.e. when a taxon found in ancient strata is recovered as derived in the phylogeny [47]. However, the very good stratigraphic congruence indices (computed using the strap v. 1.4 package in R [48]) for our resulting phylogenies indicate that this effect is probably weak in our case (see also electronic supplementary material, ESM1, ESM6 (time bins) and ESM8 (R script)).

### 2.4. Morphospaces

To analyse the evolutionary history of polycotylids, we modified the Bauplan/'ecomorphological' dataset of Fischer *et al*. [9], which uses a series of continuous measurements and ratios that collectively summarize the general Bauplan of short-necked plesiosaurians. The dataset focuses on single specimens, but, in some cases, mean values from multiple specimens have been used (see electronic supplementary material, ESM1 and ESM7). We restricted the data to Polycotylidae and we added novel data on *Thililua longicollis* and *Plesiopleurodon*, based on first-hand observations, photographs and the literature [28], and on *Dolichorhynchops herschelensis* [38], *Sulcusuchus erraini* [31] and *Mauriciosaurus fernandezi* [33], based on the literature. We also modified this dataset by removing the relative neck and relative skull lengths (which were rarely scored among polycotylids because few trunk lengths are known), and we replaced them with a ratio of the neck length over skull length, which captures the wide variations of general body shape in plesiosaurians [1,4]. We also added a character describing the snout size relative to the total mandible length, as well as the density of teeth in the symphysis. The dataset thus incorporates nine continuous characters scored for 17 polycotylid taxa (see electronic supplementary material, ESM1 and ESM7). We have collected Bauplan data on *Manemergus anguirostris* (see electronic supplementary material, tables S1–S3), whose only known specimen is a highly juvenile individual (R.B.J.B. 2010, personal observations). Some other polycotylids, which are included in our analysis, may be represented by subadult or slightly immature specimens. However, *Manemergus anguirostris* has very small body size and bears osteological indicators of young juvenile status, including the extremely incomplete ossification of girdle elements and the presence of a large cranial fontanelle. The presence of a non-osteologically mature OTU (thus exhibiting a juvenile, plesiomorphic-like morphology) in the datasets could artificially inflate the apparent disparity of Turonian polycotylids. For this reason, we removed *Manemergus anguirostris* from our analyses of morphospace occupation and disparity over time (see electronic supplementary material, table S3, and also ESM1 and ESM7 (Bauplan data) and ESM8 (R script)).

We visualized the morphological disparity of polycotylids via principal coordinate analyses of the Bauplan dataset, applying the Cailliez correction for negative eigenvalues and using the ape package (v. 4.1) [41]. We also computed a phylomorphospace using the phytools (v. 0.5-64), zoo (v. 1.7-14), paleotree (v. 2.7) and ape (v. 4.1) packages in R [41,43,49,50]. We used the most parsimonious tree from the reduced consensus analysis with the best stratigraphic congruence index (the Gap Excess Ratio here), because the phylomorphospace approach requires a fully resolved tree with branch lengths, not a consensus tree. We computed the Gap Excess Ratio score using the strap (v. 1.4) package in R [48]. We applied a completeness threshold, discarding taxa for which less than 50% of the morphological characters can be scored.

## 3. Systematic palaeontology

Plesiosauria de Blainville, 1835

Leptocleidia Ketchum & Benson, 2010

Polycotylidae Cope, 1869

*Thililua longicollis* Bardet *et al*., 2003

Figures 2–7

### 3.1. Holotype

MHNGr.PA.11710, a skull, neck and anterior dorsal region from the Lower Turonian of Goulmima, Morocco [20].

### 3.2. Emended diagnosis

Autapomorphic features among Leptocleidia: (i) posterolaterally expanded premaxilla excluding the frontal from the margin of the external naris; (ii) presence of two foramina on the jugal of different sizes (a small foramen just dorsal to the jugal–maxilla suture and a large one ventral to the jugal–postorbital suture): *Edgarosaurus muddi* possesses two small foramina of equal size on the jugal [14], *Trinacromerum bentonianum* possesses a single jugal foramen near the postorbital suture [16]; other polycotylids lack jugal foramina; (iii) lack of a splenial participation in the symphysis (unique among Polycotylidae); (iv) presence of a mesiolabial carina in the premaxillary teeth (probably as in *Manemergus anguirostris* [25]); (v) 30 cervical vertebrae (26 in *Polycotylus latipinnis* and *Edgarosaurus muddi*; 25 in *Manemergus anguirostris*; 21 in *Dolichorhynchops* spp. and 21 in *Trinacromerum bentonianum* [14,16,19,25]) and (vi) cervical centra bear lateral longitudinal ridges (also present in elasmosaurids and long-necked microcleidids and cryptoclidids ([18]; e.g. [51])).

*Thililua longicollis* can also be characterized by the combination of the following unusual features (see below for a thorough comparative description): (i) presence of a long maxillary groove anterior to the external naris (as in *Plesiopleurodon wellesi*, the ‘Richmond pliosaur’ (QM F18041) and *Sulcusuchus erraini* among polycotylids [3,28,31], plus the Early Jurassic pliosaurid *Hauffiosaurus* [52,53]); (ii) presence of small paired frontal foramina (shared with *Manemergus anguirostris* [25]); (iii) tooth row extends posteriorly up to the level of mid-orbit only (shared with *Manemergus anguirostris* [25] and *Dolichorhynchops bonneri* [16]); (iv) posterior cervical neural spine curves posterodorsally (uniquely shared with the ‘Richmond pliosaur’ (QM F18041) among Polycotylidae and otherwise widely distributed among leptocleidids and early xenopsarians [3]).

### 3.3. Type locality and horizon

Near Goulmima, Er-Rachidia Province, southern Morocco. Unit 4 of the Cenomanian**–**Turonian limestone bar. Middle Turonian, Late Cretaceous [54,55].

### 3.4. Comment on *Manemergus anguirostris*

Our diagnosis of *Thililua longicollis* indicates several features uniquely shared with another Turonian polycotylid from Morocco, *Manemergus anguirostris* (table 1). We also find several differences between the two, which are highlighted in our description below. Nevertheless, *Manemergus anguirostris* possesses several features consistent with a highly juvenile ontogenetic status, including its relatively brevirostrine snout (compared with other polycotylids), fontanelle in the skull roof (interpreted as a pineal foramen by Buchy *et al*. [25]) and highly incomplete ossification of some postcranial bones, including the coracoids (R.B.J.B. 2010, personal observations of SMNK-PAL 3861). Because of the similarities between these two taxa, O'Keefe [16] found them as sister taxa. We have problems resolving the phylogenetic position of *Manemergus anguirostris* (described below), but nevertheless find it likely that it is closely related to *Thililua longicollis* and possibly congeneric, if all of their shared features were added as new characters in the phylogenetic dataset. It is unlikely, however, that *Manemergus anguirostris* is a species-level synonym of *Thililua longicollis* because these taxa have different counts of cervical vertebrae (30 in *Thililua longicollis* compared with 25 in *Manemergus anguirostris* [20,25]).

**Table 1.** Comparison of some unusual features of *Thililua longicollis* and *Manemergus anguirostris*.

anatomical feature	*Thililua longicollis*	*Manemergus anguirostris*
paired frontal foramina	yes	yes
absence of a pineal foramen	yes	probably yes (the pineal foramen reported in Buchy *et al*. [25] is probably a fontanelle)
jugal foramina	yes	probably no
reniform orbit	yes	probably yes
carinated tooth crown	yes	yes
expanded premaxilla excluding the frontal from the external naris	yes	no
high temporal bar	yes	yes (even higher)
fontanelle	no	yes^a^
squamosal bulb	absent	present
estimated maxillary tooth count	22	9–10^a^
mesialmost premaxillary teeth contact each other mesially	yes	possibly yes
strongly reduced first premaxillary alveolus	yes	no
cervical centra	30	25
lateral ridge on cervical centra	yes	no

^a^Conditions probably resulting from osteological immaturity.

## 4. Redescription of *Thililua longicollis*

### 4.1. Note on taphonomy

**Figure 2.**
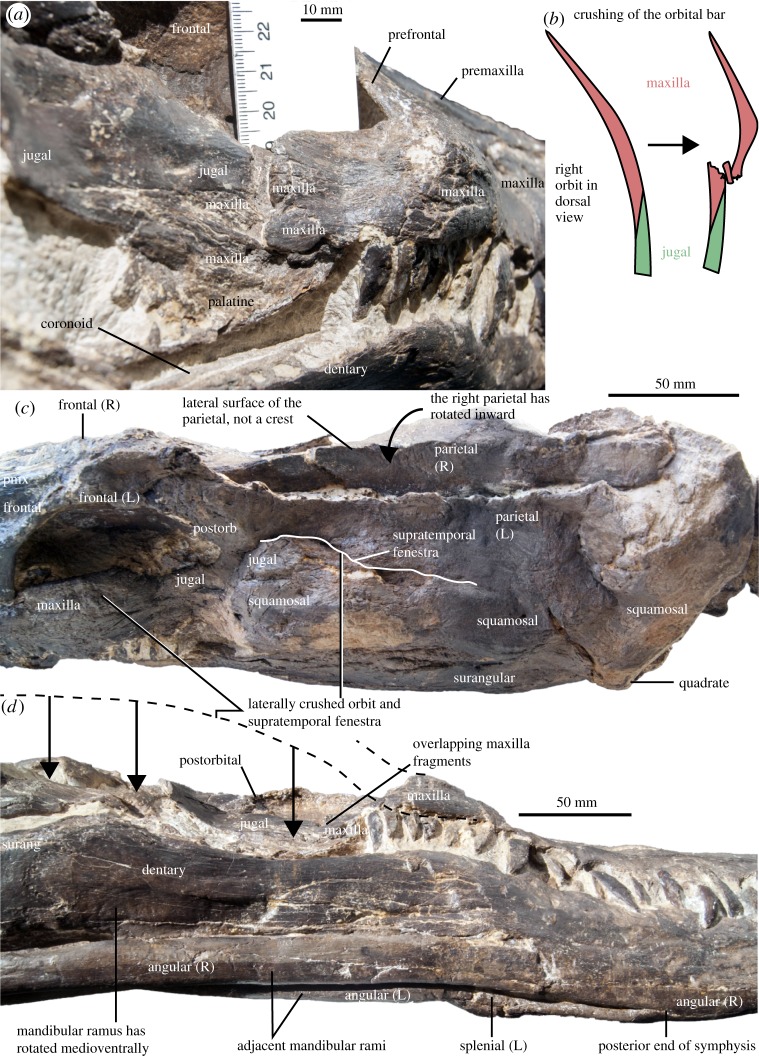
Taphonomy of the holotype skull of *Thililua longicollis* (MHNGr.PA.11710) from the middle Turonian of Morocco. (*a*) Posterolateral view of the right orbital bar showing the break and overlap due to the lateral crushing. (*b*) Illustration of the crushing of the orbital bar, in the dorsal view. (*c*) Temporal region in the dorsolateral view, showing the complete mediolateral crushing of the supratemporal fenestra and medioventral rotation of the right parietal. (*d*) Skull in the ventrolateral view, showing the crushing of the orbital bar and of the mandibular rami, which are now adjacent.

**Figure 3.**
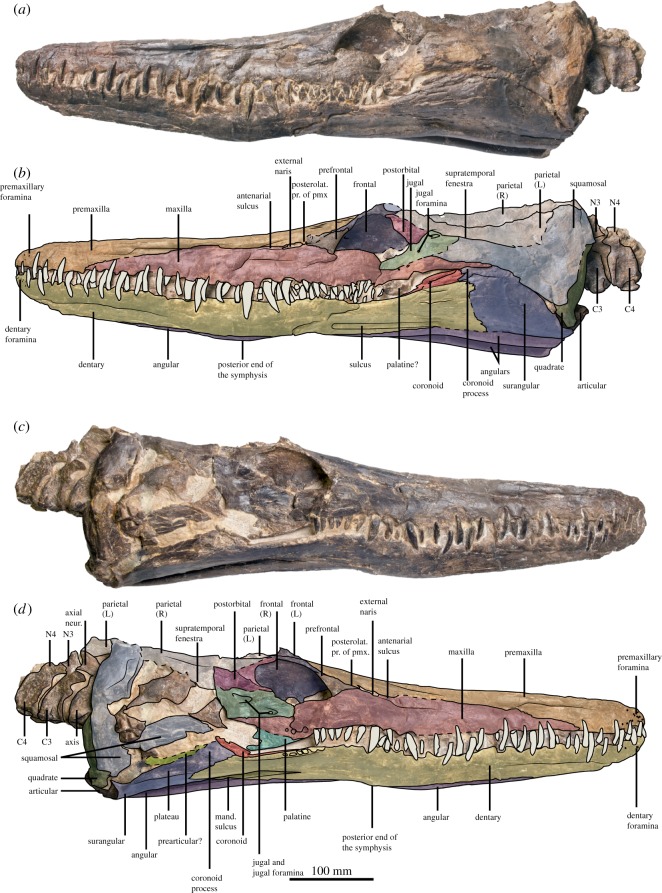
Skull, mandible and anterior cervical centra of the holotype of *Thililua longicollis* (MHNGr.PA.11710) from the middle Turonian of Morocco, in left lateral (*a*,*b*) and right lateral (*c*,*d*) views. (*b*,*d*) Osteological interpretations of (*a*,*c*), respectively.

**Figure 4.**
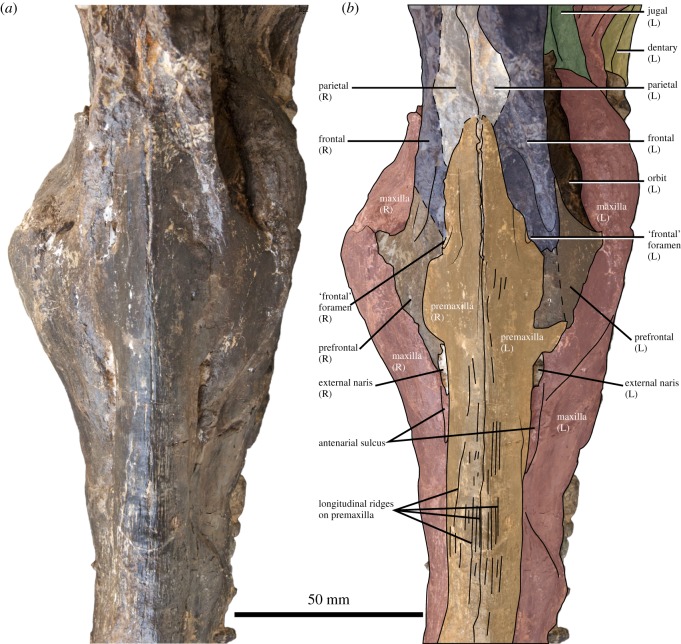
Rostrum and frontal region of the holotype of *Thililua longicollis* (MHNGr.PA.11710) from the middle Turonian of Morocco. (*a*) Dorsal view and (*b*) osteological interpretation of (*a*).

**Figure 5.**
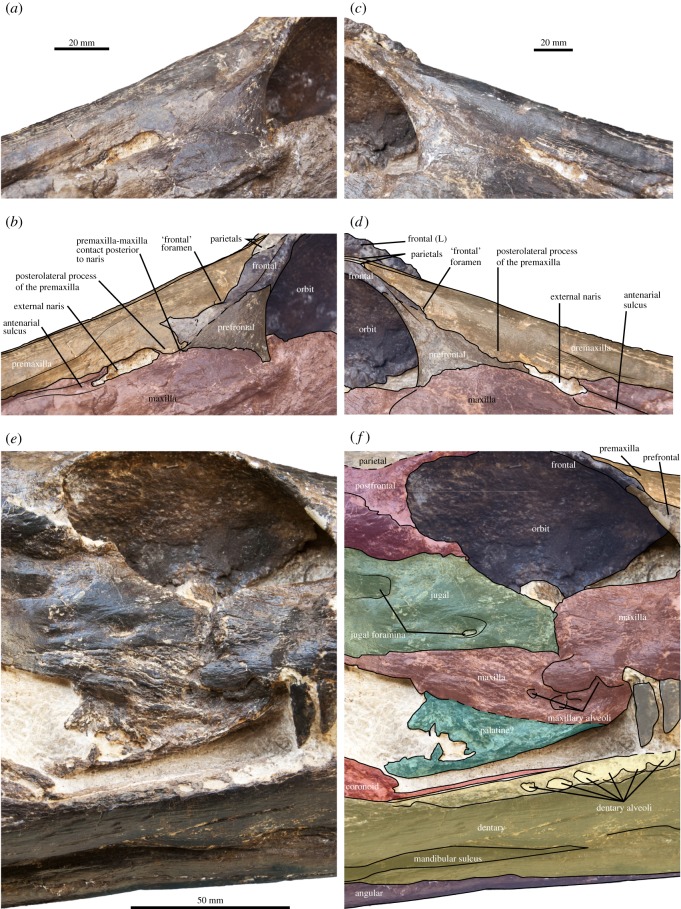
Narial and orbital region of the holotype of *Thililua longicollis* (MHNGr.PA.11710) from the middle Turonian of Morocco. (*a*,*b*) Left external naris in the anterolateral view (osteological interpretation in (*b*)). (*c*,*d*) Right external naris in the anterolateral view (osteological interpretation in (*d*)). (*e*,*f*) Right orbit and orbital bar in the lateral view, showing parts of the broken maxilla and palatine (osteological interpretation in (*f*)).

**Figure 6.**
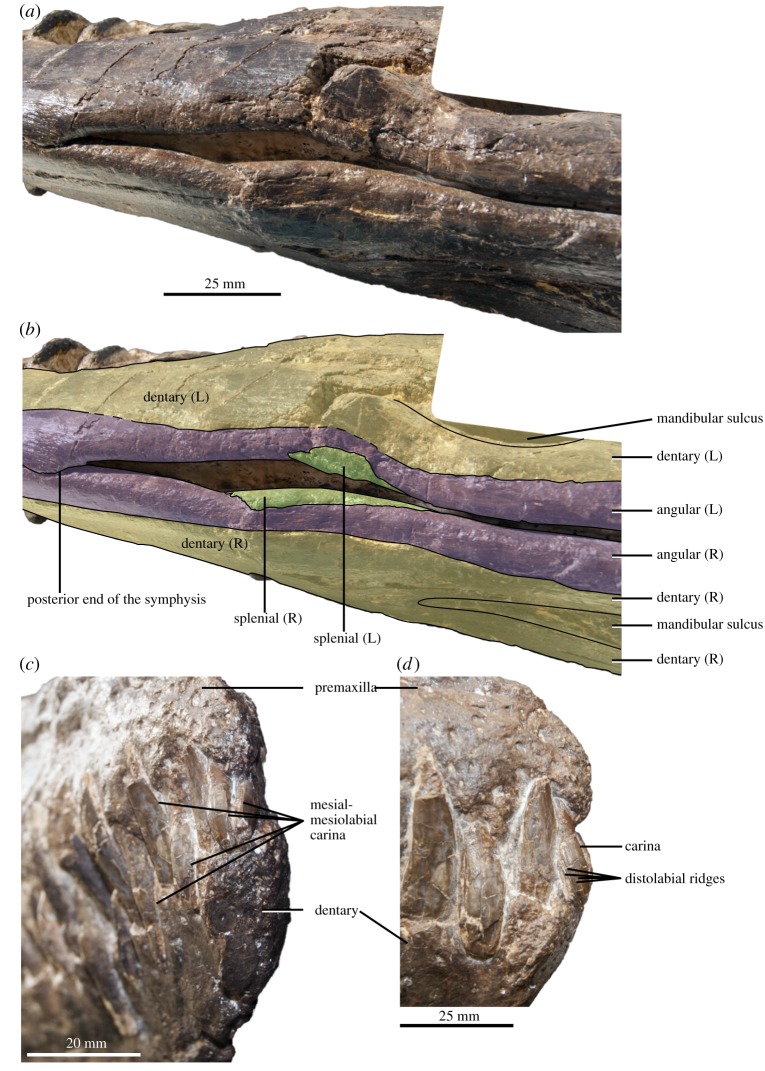
Mandible and teeth of the holotype of *Thililua longicollis* (MHNGr.PA.11710) from the middle Turonian of Morocco. (*a*,*b*) Post-symphysis region of the mandible in the ventral view (osteological interpretation in (*b*)). (*c*,*d*) Morphology of mesial premaxillary and dentary teeth. Note the mesial or mesiolabial carinae and the distolabal ridges on these teeth. (*c*) Snout tip in the right anterolateral view. (*d*) Snout tip in the right lateral view.

MHNGr.PA.11710, the holotype and only reported specimen of *Thililua longicollis*, is strongly crushed laterally (figures 2–6). This crushing particularly affects the orbital and temporal bars, resulting in an unnatural contact between the maxilla and the jugal. Indeed, the maxilla now vastly overlaps the anterior part of the jugal, especially on the right lateral side (figures 2 and 5). This is probably what led Bardet *et al*. [20] to interpret the large bone forming the posteroventral margin of the orbit as part of the maxilla, while we regard this as the jugal. This, in turn, requires reinterpretation of the entire postorbital and temporal bars, as well as the interorbital skull roof. The crushing of the holotype has also rotated the ventral margins of the mandibles medially, while the ventral margin of the squamosal arch has rotated anteriorly and the arch has rotated in the dorsoventral plane (figures 2 and 3). Because of this distortion, some of the features of *Thililua longicollis*, notably the shape of its rostrum and mandible, should not be used to differentiate *Thililua longicollis* from other forms (contra [33]). See table 2 for a series of measurements on the holotype.

**Table 2.** New and revised cranial measurements of the holotype of *Thililua longicollis* (MHNGr.PA.11710). All measurements are in mm and were taken using a calliper.

skull length	665
preorbital length^a^	392.5
postorbital length^a^	197.5
maxilla length	460
external naris length	23
frontal foramen length	10
jugal foramen length	15
orbit length	82
orbit height	50
supratemporal fenestra length^a^	112.5
symphysis length	290
mandible height at the level of the coronoid eminence^a^	82.5
first alveolus diameter	5
third alveolus diameter	10

^a^Measurements that are the average of the values obtained from the left and right sides of the skull are shown.

### 4.2. Rostrum

The cranium of MHNGr.PA.11710 is 665 mm long and the rostrum is 392.5 mm long (taken as a mean from the left and right lateral sides), giving a preorbital ratio (rostrum length/mandible length) of 0.59. The rostrum is thus slightly shorter than that of most other polycotylids: this ratio is 0.65 in *Polycotylus latipinnis*, and up to 0.69 in *Pahasapasaurus haasi* and in *Dolichorhynchops osborni* [25,56]. The only polycotylids with proportionally smaller rostra than that of *Thililua longicollis* are *Edgarosaurus muddi* and *Manemergus anguirostris* (0.49) [14,25]. The rostrum is 130 mm high just anterior to the orbits, giving a rostrum shape ratio of 3.02 (this ratio is overestimated due to lateral crushing of the specimen; figures 3 and 4). The alveolar margin is straight in lateral view. The slight curvature of the ventral margin of the maxilla between the external naris and the orbit is also probably an effect of the severe lateral crushing of the specimen, as both the suborbital and temporal bars have displaced medially and slightly dorsally into the orbits and supratemporal fenestra, respectively. The weak embayment of the temporal bar appears genuine, however.

### 4.3. Premaxilla

The premaxilla bears a series of small foramina on its anterior surface as in *Pahasapasaurus haasi* and *Polycotylus latipinnis* [19,56]. Five premaxillary alveoli are present, as in most polycotylids [14,15,19,25,30] except *Pahasapasaurus haasi* where there are six [56], and six to seven in *Edgarosaurus muddi* [14]. The first alveolus is strongly reduced in diameter (50% smaller than the third one). This morphology is widespread among plesiosauroids and is present in some polycotylids, such as *Manemergus anguirostris* (SMNK-PAL 3861), *Pahasapasaurus haasi* [56], *Palmulasaurus quadratus* [15] and *Dolichorhynchops herschelensis* (RSM P2310). However, it is absent in others, including *Edgarosaurus muddi* (MOR 751) and *Polycotylus latipinnis* [19]. The first premaxillary teeth contact each other mesially, a feature of uncertain distribution that is also present in *Dolichorhynchops herschelensis* (RSM P2310). The premaxilla–maxilla suture is weakly crenulated and is gently oblique compared with the alveolar margin; the suture does not rise abruptly but rather progressively, unlike in *Pahasapasaurus haasi* [56]. No diastema is visible at the premaxilla–maxilla suture. The posteromedial process of the premaxilla forms the dorsal margin of the external naris (figures 3–5). This is distinct from all other polycotylids that we have examined, in which the premaxilla is prevented from contacting the external naris by contact between the maxilla and frontal (e.g. [14,16,19,38]). Participation of the premaxilla in the dorsal margin of the external naris was illustrated by Carpenter [28] in *Plesiopleurodon wellesi*, although we have not been able to confirm this from our notes. Posterior to the external naris, the lateral portion of the premaxilla expands laterally, forming a sheet-like flange that partially covers the prefrontal and the frontal, and excludes the frontal from the external naris in exterior view (a unique feature of *Thililua longicollis*). The posterior process of the premaxilla is textured by a series of longitudinal grooves (figure 4), reminiscent of the condition seen in many pliosaurids [57–59] and the polycotylids *Dolichorhynchops tropicensis* [30] and *Dolichorhynchops osborni* [60], although these grooves are shallower and more superficial in *Thililua longicollis*. Furthermore, this region in *Thililua longicollis* is predominantly occupied by a transversely narrow median ridge that is more widespread among xenopsarians, including most polycotylids, such as *Manemergus anguirostris* (SMNK-PAL 3861), *Pahasapasaurus haasi* [56] and *Plesiopleurodon wellesi* [3]. Slightly more posteriorly, the premaxilla forms the anterior and dorsal margins of a small and slit-like foramen, corresponding to the ‘frontal foramina’ of Bardet *et al*. [20]. The premaxilla probably contacts a thin anterior process of the parietal and this contact is located at the level of mid-orbit.

### 4.4. Maxilla

The maxilla bears 22 alveoli (on the right; only 20 are preserved on the left side), which is on the lower end in polycotylids, but much more than the 9 to 10 alveoli counted in *Manemergus anguirostris* [25]. Nevertheless, there is evidence that the maxillary alveolar count can increase during the ontogeny of longirostrine plesiosaurs [61]; it is thus possible that this difference occurs, at least in part, due to the young juvenile status of the holotype and only reported specimen of *Manemergus anguirostris*. Maxillary teeth of *Thililua longicollis* are more widely spaced than premaxillary teeth; thick bony walls separate each alveolus. Posterior to the level of the external naris, alveoli progressively reduce in diameter and become more closely spaced. The tooth row extends posteriorly up to the level of mid-orbit, as in *Manemergus anguirostris* [25], *Dolichorhynchops bonneri* [16] and *Dolichorhynchops osborni* [5,60], but unlike in some other polycotylids, including *Edgarosaurus muddi* and *Polycotylus latipinnis*, in which the tooth row extends nearly up the posterior end of the orbit [14,19]. The post-alveolar portion of the maxilla of *Thililua longicollis* rapidly decreases in dorsoventral height and forms a posteriorly tapering process located ventrally to the jugal and the squamosal. This differs from the situation in other polycotylids, in which the maxilla reduces its dorsoventral height from a more anterior location, right at the anterior margin of the orbit. This gives the orbit a large, suboval shape (e.g. *Trinacromerum bentonianum* and *Polycotylus latipinnis*) [16,19]. *Thililua longicollis* differs further from the morphology of other polycotylids because the maxilla extends into the anteroventral portion of the orbit as a prominent convexity, forming a sharp angle with the posterior margin of the prefrontal, and giving the orbit a reniform shape. This unusual orbit shape, as evident in the left orbit of the holotype specimen (MHNGr.PA.11710), is otherwise seen in *M. anguirostris* [25] and elasmosaurids [22]. *Plesiopleurodon* [28] and the ‘Richmond pliosaur’ (QM F18041) also have maxillae that are dorsoventrally thick ventral to the orbit compared with other polycotylids, but do not possess reniform orbits. The maxilla of *Thililua longicollis* terminates posteriorly, as seen in lateral view, at the level of the coronoid process, substantially underlapping the ventral margin of the squamosal, as in other polycotylids [14,16,19]. A long groove extends anteriorly from the external naris along the dorsolateral surface of the maxilla (‘antenarial groove’ in figures 3–5). This is evident in spite of the lateral crushing of the specimen and is developed to a similar extent as those seen in *Plesiopleurodon wellesi*, the ‘Richmond pliosaur’ (QM F18041; [3,28]) and *Sulcusuchus erraini* [31,62]. A similar groove is also seen outside of Polycotylidae, in the early pliosaurid *Hauffiosaurus* [52,53].

### 4.5. Prefrontal

The prefrontal is triangular, bearing a long, thin anterior process that contacts the posterior margin of the external naris; this narial process appears much thinner than in other polycotylids such as *Edgarosaurus* [14], *Dolichorhynchops* spp. [16,38] and *Polycotylus latipinnis* [19]. The anterolateral surface of the prefrontal is slightly saddle-shaped. The prefrontal forms a weakly crenulating suture with the maxilla ventrally and is partially covered by the posterolateral flange of the premaxilla. The prefrontal possesses a tubular posterodorsal process forming the anterior margin of the orbit and contacting the frontal via an oblique suture. The contribution of the prefrontal to the orbital rim is thus larger than in *Dolichorhynchops bonneri* [16] and resembles that of *Edgarosaurus* and *Dolichorhynchops herschelensis* [14,38].

### 4.6. Frontal

The frontal of *Thililua longicollis* has a reduced external exposure. It is excluded (externally at least) from the margin of the external naris by the posterolateral flange of the premaxilla; this condition appears unique to *Thililua longicollis* (and perhaps *Plesiopleurodon*, but the quality of preservation of this region in the type and only specimen of this taxon is poor [28]). The frontal is thus splint-like and forms the dorsal margin of the orbit. Its contacts with the parietal and the postorbital are hardly discernible. Internally, the frontal forms an extensive, concave ventromedial process socketing the eyeball as in *Pahasapasaurus haasi* [56]; this concave surface is pierced by two foramina. A foramen pierces the dorsal surface of the frontal in *Thililua longicollis*. This foramen is situated along the frontal – premaxilla contact, dorsal to the prefrontal–frontal suture. Both left and right foramina are 10 mm long and appear uniquely present in *Thililua longicollis* and *M. anguirostris* [20,25] among polycotylids. The frontal foramen does not contact the premaxilla in *Manemergus* because this taxon lacks a posterolateral process of the premaxilla. A larger and more elongated ‘frontal fenestra’ wedged between the frontal and the prefrontal has been described in *Dolichorhynchops osborni* and *Dolichorhynchops herschelensis* [28,38] and might also be present in *Plesiopleurodon wellesi* [28].

### 4.7. Note on the orbital region

We reinterpreted the orbital region of *Thililua longicollis*. Bardet *et al*. [20] described an extensive posterior portion of the maxilla, forming the posterior margin of the orbit and leaving only a narrow space dorsally for an extremely reduced jugal and a postorbital that would be excluded from the supratemporal fenestra [20]. This interpretation of the morphology would be highly distinct from other polycotylids and from most other plesiosaurians. However, it resulted from difficulties interpreting the skull, which has undergone strong lateral compression. Close inspection indicates that the maxilla actually decreases in dorsoventral height just posterior to the level of the distalmost maxillary alveoli. It then forms a long, posteriorly tapering tongue-like process underlapping the jugal and the squamosal ventrally, similar to the condition seen in other polycotylids such as *Dolichorhynchops bonneri* and *Polycotylus latipinnis* [16,19]. Because of the strong lateral crushing, the maxilla–jugal contact has been broken and, on both sides, the maxilla has been broken, forming bony sheets that overlap each other and that also overlap the anterior portion of the jugal. The crack situated at mid-orbit on the suborbital bar (figures 2–5) is thus an unnatural maxilla–jugal contact. The jugal has a large lateral extension, as does the postorbital, and our revised interpretation of the postorbital bar closely resembles the architecture seen in other polycotylids (e.g. [14,16]).

### 4.8. Jugal

The jugal is dorsoventrally thick and elongated, similar to the morphology of many other plesiosaurians and the polycotylids *Edgarosaurus muddi* [14] and the ‘Richmond pliosaur’ (QM F18041). This morphology is probably plesiomorphic for Polycotylidae and differs from the condition seen in polycotylines, such as *Trinacromerum bentonianum*, *Dolichorhynchops* spp. and *Polycotylus latipinnis*, which have reduced jugals [16,19,60]. The presence of a large jugal, combined with the relatively large dorsoventral thickness of the maxilla ventral to the orbit, is that *Thililua longicollis* possesses a proportionally smaller orbit than that of many other polycotylids. A large lateral foramen is present 10 mm ventral to the jugal–supraorbital suture, as in *Trinacromerum bentonianum* [16]. Another, smaller foramen is present, dorsal to the maxilla–jugal suture. The presence of two jugal foramina has only been reported in *Edgarosaurus muddi* [14], but jugal foramina are of equal size in this taxon; and more than two jugal foramina are present in the ‘Richmond pliosaur’ (QM F18041). Preservation of bone surfaces in *Manemergus anguirostris* is poor in this region, so it is not possible to determine whether foramina are present (SMNK-PAL 3861). The jugal foramina of *Thililua longicollis* are present on both sides, but are harder to discern on the left lateral side because of the strong crushing of the suborbital bar on that side.

### 4.9. Postorbital

The postorbital forms a thick posterior process, resulting in a dorsoventrally thick postorbital bar, unlike in *Edgarosaurus muddi*, *Dolichorhynchops* spp., *Plesiopleurodon wellesi* and *Polycotylus latipinnis* where this bar is reduced to a thin arch [14,16,19,28,38]. The only other polycotylid with a dorsoventrally thick postorbital bar is *Manemergus anguirostris*, where its dorsal edge is nearly as high as the skull table [25], thus thicker than in *Thililua longicollis*.

### 4.10. Temporal region and squamosal arch

This region is severely crushed laterally in the holotype of *Thililua longicollis* and thus yields few useful characters. The parietal crest is not visible on the right lateral side (contra Bardet *et al*. [20]). This crest itself rises dorsally only to the approximate level of the skull table, similar to the condition in *Edgarosaurus muddi* [14], *Plesiopleurodon wellesi* [3,28] and *Manemergus anguirostris* [25], but unlike in polycotylines, which have taller parietal crests [16,19,38,60]. *Thililua longicollis* lacks a fontanelle between the parietals and the squamosals, unlike in *M. anguirostris* (where it was described as a pineal foramen that would be located medially, at the parietal–squamosal contact [25]). However, the presence of this fontanelle in *Manemergus anguirostris* is probably due to the young juvenile ontogenetic stage of the specimen [3] (table 1). As in *Manemergus anguirostris* [25] and most other polycotylids [14,16,19], *Thililua longicollis* lacks a pineal foramen. *Edgarosaurus muddi* is the only polycotylid to possess a definite pineal foramen [14], which is probably plesiomorphic given the phylogenetic position of this taxon (some geologically younger polycotylids have a narrow slit-like opening that has sometimes been regarded as a pineal foramen; e.g. [19]). The parietal–squamosal contact of *Thililua longicollis* is hardly discernible due to crushing of the squamosals, obscuring the original orientation of their dorsal processes. The quadrate has a bulbous shaft and is slightly constricted dorsal to the glenoid surface.

### 4.11. Palate

Only a small putative fragment of the palate is visible, attached to the broken posteroventral surface of the end of the dentigerous portion of the maxilla on the right lateral side. Owing to its position, this might be a fragment of the palatine or the ectopterygoid boss.

### 4.12. Dentary

Small foramina cover the anterior surface of the dentary close to the symphysis (figure 6). As in the premaxilla–maxilla, the spacing of teeth varies slightly along the dentary: the three mesialmost teeth are closely spaced, resulting in a tightly interlocking pattern with the premaxillary teeth. This might be an effect of the lateral compression of the rostrum, as this region was originally slightly curved. Alveoli are more widely spaced posteriorly (but much less than in *Manemergus anguirostris* [25]). The alveoli start decreasing in diameter by the level of the anterior margin of the orbit. In that region, the teeth are closely spaced: the interdental plates are 1–2 mm wide mesiodistally. The dentary tooth row is located slightly higher dorsally than the level of the glenoid fossa, as in other polycotylids, including *Edgarosaurus muddi*, *Plesiopleurodon wellesi*, *Manemergus anguirostris* and *Dolichorhynchops bonneri* [14,16,25,28]. Posterior to the tooth row, the dentary forms a bony sheet covering the surangular laterally; the dentary extends posteriorly up to the level of the coronoid eminence, but most of that process is formed by the surangular, as in other polycotylids [3,19,25,38].

### 4.13. Coronoid

The coronoid is poorly preserved as a broken bony sheet covering the medial surface of the dentary at the level of the orbit and the dorsomedial surface of the coronoid eminence, as in other polycotylids [14,19] (figures 3 and 5).

### 4.14. Surangular

The surangular forms, with the coronoid anteromedially, most of the coronoid eminence. Posteriorly, the surangular rapidly decreases in dorsoventral height and then forms a dorsoventrally oriented, crescent-shaped process that participates to the anterior part of the glenoid cavity (figure 3). This condition differs from *Dolichorhynchops bonneri* where the entire glenoid cavity appears to be formed by the articular [16].

### 4.15. Angular

The angular forms a long anterior process extensively participating in the symphysis, as in most other polycotylids, and extending anteriorly by more than one-third of the symphysial length, as in *Manemergus anguirostris* and *Polycotylus latipinnis* [19,25] (figure 3). This is unlike the condition in *Eopolycotylus rankini* and the ‘Richmond pliosaur’ (QM F18041), in which the angular falls short of participating in the symphysis [15]. Posterior to the level of mid-orbit, the angular slowly rises dorsally, via an oblique suture with the dentary. Robust, anteroposteriorly oriented buttresses are present on the posteroventral part of the dentary and the dorsolateral surface of the angular. These buttresses form the dorsal and ventral bounds of a wide, anteroposteriorly oriented sulcus that occupies essentially the entire posterior third of the lateral surface of the mandible. This is present in most leptocleidians as well as in rhomaleosaurids [3,63]. Posterior to the coronoid eminence, the angular of *Thililua longicollis* progressively decreases in dorsoventral height.

### 4.16. Splenial

Unusually among polycotylids, the splenial has a markedly reduced ventral exposure (figure 6), only briefly appearing as an elongated bony sheet covering the ventromedial surface of the angular for 3–4 cm and vanishing at least 3 cm before the posterior margin of the symphysis. Therefore, the splenial does not contribute to the symphysis ventrally in *Thililua longicollis*, although it may have continued further anteriorly internally within the mandible. This markedly contrasts with other polycotylids, including *Manemergus anguirostris*, in which the splenial always contributes to the symphysis in ventral view [15,19,25]. In *Mauriciosaurus fernandezi*, the splenial appears extremely extensive, forming most of the ventral surface of the mandibular rami [33]. The fragmentary mandible of *Palmulasaurus quadratus* has a pair of wedge-shaped bones that were interpreted, by default, as splenials [15]. However, the architecture of the symphysis in *Thililua longicollis* questions this assumption, as these bones could also be interpreted as angulars. Reported specimens of other ‘palmulasaurines’ do not help to clarify the condition in *Palmulasaurus*, as they also do not preserve the relevant bones of the mandible in sufficient completeness [56,64]. More complete specimens are therefore needed to identify the symphysial bones unambiguously in *Palmulasaurus*.

### 4.17. Articular

Only the anterior portion of each articular is preserved, lacking the retroarticular process. It forms a thick cup-like process forming the posterior and posteroventral part of the glenoid. It is V-shaped in mediolateral cross-section.

### 4.18. Dentition

The dentition is relatively homodont, with a progressive increase in tooth size distally as far as the anterior part of the maxilla. Teeth become smaller and bulkier posterior to the level of the external naris. The first premaxillary tooth is reduced and contacts the corresponding tooth from the other side mesially along most of its length. However, this extended contact of the mesialmost teeth might be an artefact of the strong lateral compression of the rostrum. Unusually, a mesiolabial carina is present in premaxillary and anteriormost dentary teeth (figure 6). A ‘faint rostral carina’ has also been described in *Manemergus anguirostris* [25] and a ‘weak posterior’ carina is present in *Eopolycotylus rankini* [15]. ‘Subtle’ anterior and posterior carinae are present in *Polycotylus latipinnis* [19]. Crowns appear smooth apically and along their mesial and labial surfaces (as in *Eopolycotylus rankini* and *Dolichorhynchops bonneri* [15,16]), but bear coarse ridges on their distal and distolabial surfaces (lingual surfaces are not available for description). This differs from *Palmulasaurus quadratus*, *Dolichorhynchops tropicensis* and *Polycotylus latipinnis*, in which the entire diameter of at least the basal third of the crown bears fine ridges [15,19,30,60]. In *Sulcusuchus erraini*, ridges are present along the entire diameter of the crown except the ‘anterior’ (presumably mesial or mesiolabial) surface, but there is also an alternating pattern of ridged and non-ridged tooth crowns along the jaw [31]. Teeth are elongated (2.69 times apicobasally longer than basally thick, but relatively smaller than in *Manemergus anguirostris*) and slightly recurved, differing from the stout teeth of *Eopolycotylus rankini*, *Plesiopleurodon wellesi*, *Dolichorhynchops bonneri*, *Polycotylus latipinnis* and *Edgarosaurus muddi* [14–16,19,28]. Many of the large teeth from the anterior third of the rostrum part are not genuine and have been reconstructed in plaster and painted.

### 4.19. Atlas–axis

The atlas–axis complex is not fully prepared and most of the atlas is hidden by the posterolateral margin of the cranium. The shape of the axial rib facet cannot be determined with precision, but it articulates solely with the axis, a condition only otherwise seen in *Edgarosaurus muddi* in polycotylids [3,14]. A large hypophyseal (ventral) ridge extends along the whole of the preserved length (the entire axis and the posterior part of the atlas).

### 4.20. Axial skeleton

**Figure 7.**
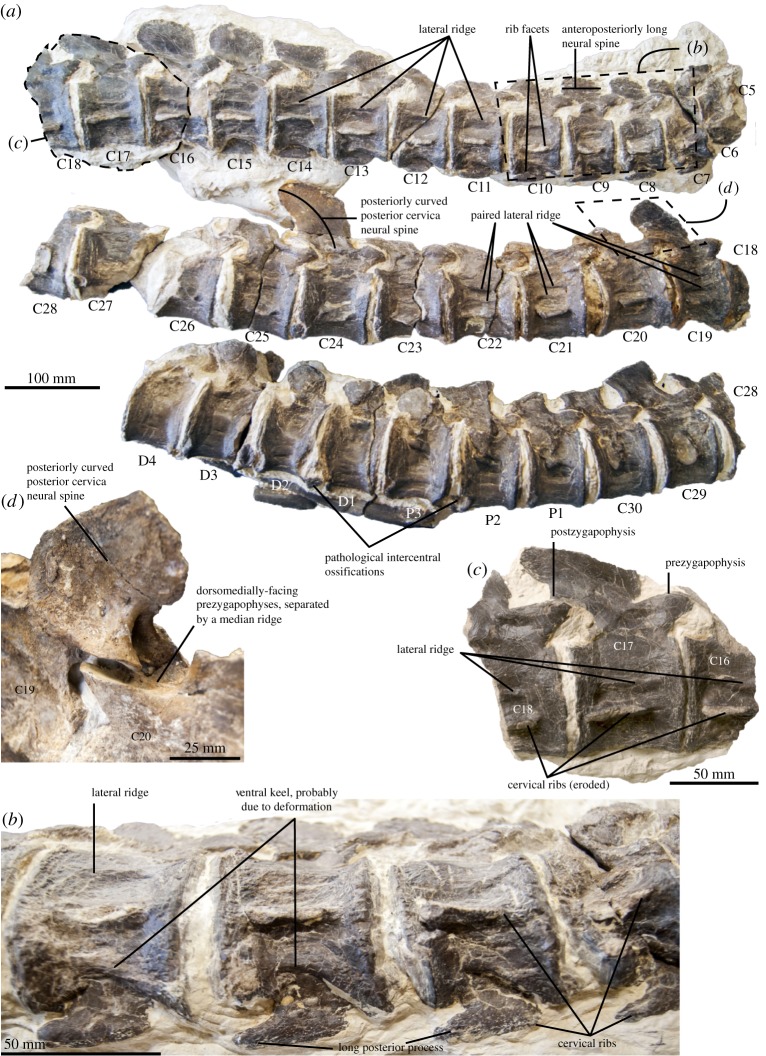
Axial skeleton (cervical and pectoral regions) of the holotype of *Thililua longicollis* (MHNGr.PA.11710) from the middle Turonian of Morocco. (*a*) Preserved axial skeleton in the right lateral view. Note the 30 cervical vertebra, the lateral ridges (single or paired) and the shape of the neural spines. (*b*) Detail of the anterior cervical region (C7–C10). Note the ventral keel and the lateral ridge. (*c*) Detail of the mid-cervical region (C16–C18). (*d*) Detail of mid-cervical neural spines (C19–C20), showing the dorsomedially facing prezygaphyses, separated by a median ridge.

Taking into account the atlas and the axis, we counted a total of 30 cervical vertebrae (figure 7), as did Bardet *et al*. [20], which is a much larger number than in other polycotylids (26 in *Edgarosaurus muddi* and *Polycotylus latipinnis*, 25 in *Manemergus anguirostris* and fewer than 23 in other polycotylids where the number of cervicals is known unambiguously [14,19]; R.B.J.B. 2010 & H.F.K. 2006, personal observations [25,28,30]). The total neck length of *Thililua longicollis*, as preserved, is 2.17 m long. This gives a neck length/skull length ratio of 3.26 for *Thililua longicollis*, while all other polycotylids range from 0.92 (*Mau. fernandezi*) to 1.81 in *Polycotylus latipinnis* (see electronic supplementary material, tables S2 and S3). This ratio reaches 1.94 in *Manemergus anguirostris*. However, this taxon is based on a juvenile individual that we regard as possibly belonging to the genus *Thililua* (table 1). The cervical centra of *Thililua longicollis* appear strongly waisted in ventral view. This shape is, however, probably exaggerated by the taphonomic and diagenetic history of the specimen and, hence, height/length ratios should not be taken at face value. These deformations render difficult any quantitative comparison of the shape of cervical centra with other polycotylids, but if we assume that the intercentrum facets were subcircular *in vivo*, then the cervical centra of *Thililua longicollis* were longer anteroposteriorly than wide mediolaterally (contra [20]). *Thililua longicollis* thus resembles *Edgarosaurus muddi*, *Plesiopleurodon wellesi*, *Pahasapasaurus haasi*, *Manemergus anguirostris* and *Dolichorhynchops tropicensis* in having cervical centra that are not shorter anteroposteriorly than their dorsoventral height [3,14,25,30,56]. The elongated neck of *Thililua longicollis* was mainly achieved by an increase of cervical elements (probably through somitogenesis) rather than a notable elongation of cervical elements (differential growth; see Soul & Benson [4] for a review of these effects in all sauropterygians), but both processes probably contributed, as is the case in elasmosaurids [35,59].

The lateral surfaces of the cervical centra bear elongate, anteroposteriorly oriented, raised ridges that taper anteriorly and posteriorly but do not reach the centrum margins (figure 7). This ridge is evident in anterior cervical centra and is located approximately 10 mm dorsal to the rib facet and is approximately 5–7 mm long mediolaterally. In more posterior cervical centra, the lateral ridge decreases in mediolateral height as the rib facet progresses dorsally. No protruding lateral ridge can be seen in the posteriormost cervical centra, where the rib facet is located dorsal to the mid-height of the centrum. Instead, paired, anteroposteriorly elongated ridges are present in centra 19–22 [20]. Among sauropterygians, such a lateral ridge has been reported in extremely long-necked forms such as microcleidids (except *Microcleidus melusinae*; [65]), the cryptoclidids *Muraenosaurus* and *Spitrasaurus*, and elasmosaurids [3,18,51,66,68], suggesting convergent adaptations among distantly related xenopsarians.

Some cervical zygapophyses are excellently preserved (figure 7). Each zygapophyseal surface faces dorsomedially and their combined width appears much smaller than the diameter of their corresponding centrum. The prezygapophysial surfaces are in close contact along most of their length, and this contact is marked by a shallow median ridge. *Thililua longicollis* shares with *Eopolycotylus rankini* and the ‘Richmond pliosaur’ (QM F18041) the possession of posterior cervical neural spines that are curved posterodorsally ([3,15]; H.F.K. 2006, personal observations). However, *Thililua longicollis* is unique in combining these features with the presence of straight, posterodorsally pointing anterior cervical neural spines. A relatively shallow dorsoventral cleft is present on the posterior surface of the last cervical and first pectoral neural spines and absent in all others where it is visible. This differs from deep clefts that are present on the anterior and posterior surfaces of the posterior cervical and the dorsal neural spines of some other plesiosaurians, including the early xenopsarian *Brancasaurus* [27], elasmosaurids [6] (char. 121,22), and the polycotylids *Eopolycotylus*, *Polycotylus* and the ‘Richmond pliosaur’ (QM F18041) ([15]; personal observations by P.S.D. and H.F.K). We therefore scored this character state as absent (char. 170.0) in our cladistic dataset. The anterior cervical neural spines of *Thililua longicollis* are longer anteroposteriorly than tall dorsoventrally, which is unique among Polycotylidae but frequently observed in elasmosaurids [3,18,59], providing another case of convergence between these taxa. Ossified elements are present between cervicals. These appear pathological and have resulted in deformations of the margin of the centra, as already noted by Bardet *et al*. [20].

## 5. Results

### 5.1. Phylogeny

**Figure 8.**
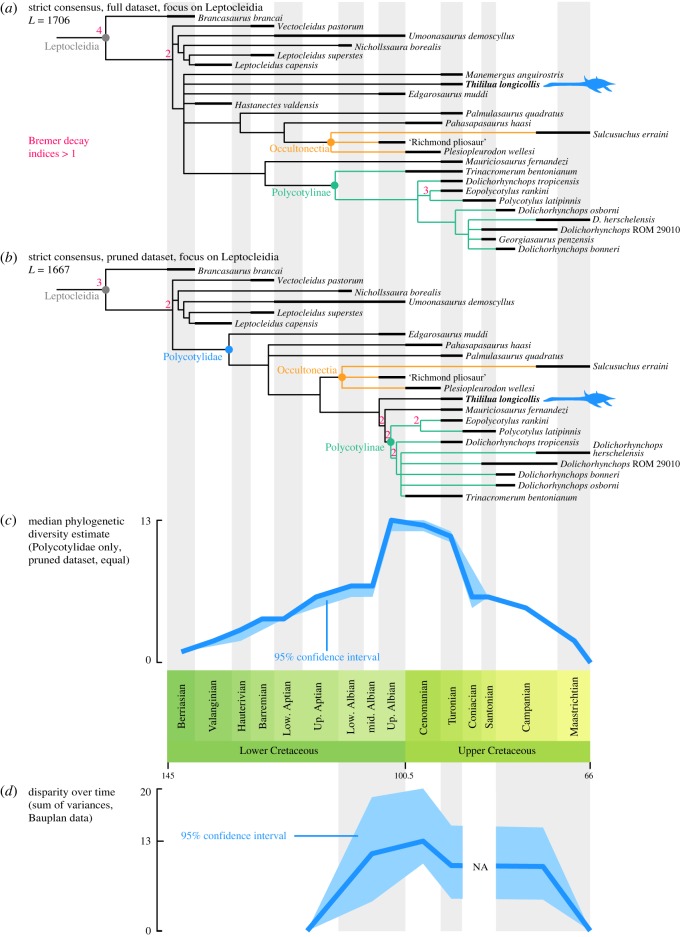
Phylogenetic relationships and diversification of leptocleidian plesiosaurians. (*a*) Focus on Leptocleidia from the strict consensus of the 50 000 most parsimonious trees arising from the maximum-parsimony analyses of the full dataset. Each tree has a length of 1706 steps. *Thililua longicollis* is indicated by the blue outline. (*b*) Focus on the Leptocleidia clade from the strict consensus of the 3584 most parsimonious trees arising from the maximum-parsimony analyses of the pruned dataset. Each tree has a length of 1667 steps. *Thililua longicollis* is indicated by the blue outline. None of the analyses yielded resampling values (bootstrap and jackknife) above 50% for leptocleidian clades and were thus not indicated. (*c*) Median phylogenetic diversity estimates using all the most parsimonious trees from the analysis of the pruned dataset in ‘equal’ time scaling, with 95% confidence interval. (*d*) Evolution of the disparity of polycotylids over time, based on the sum of variance using the Bauplan data. The time bins are wider for the sum of variances than for (*a*–*c*).

Our phylogenetic analysis recovered 50 000 trees (the limit that was set prior to the analyses), each with a length of 1706 steps (figure 8; electronic supplementary material, figure S1). The rescaled consistency index is 0.1639, indicating high degrees of homoplasy, which is well known in plesiosaurians [1,7,9]. The trees have a very good fit to stratigraphy (electronic supplementary material, ESM1 and figure S2). Leptocleidians are quite well resolved in the strict consensus tree. *Brancasaurus* is the earliest-branching leptocleidian. Polycotylids form a clade with the short-necked, but fragmentary earliest Cretaceous plesiosaur *Hastanectes valdensis* [27]. We find it likely that the recovery of this clade results from the convergent evolution of short necks and highly incomplete knowledge of the anatomy of *Hastanectes*. This hypothesis is supported by our finding that polycotylid phylogeny is better resolved and has a better fit to stratigraphy when *Hastanectes* is excluded (described below). When our full dataset is analysed, this clade is poorly resolved at its base, forming a basal polytomy among non-leptocleidid leptocleidians that unites *Hastanectes valdensis*, *Manemergus anguirostris*, *Thililua longicollis*, *Edgarosaurus muddi* and two well-resolved clades of more derived polycotylids. One of these clades represents a slightly expanded version of Polycotylinae *sensu* Albright *et al*. [15], incorporating *Trinacromerum bentonianum*, *Dolichorhynchops* spp., *Eopolycotylus*, *Polycotylus latipinnis* and *Georgiasaurus penzensis*. *Mauriciosaurus fernandezi* is recovered as the sister taxon to Polycotylinae. *Dolichorhynchops* is not recovered as a monophyletic entity: *D. tropicensis* is recovered as a relatively early branching polycotyline; the other species of the genus are paraphyletic as *Georgiasaurus penzensis* forms a clade with all other species of *Dolichorhynchops*. The other clade recovered in this analysis contains Palmulasaurinae, but is recovered here as a grade at the base of a *Plesiopleurodon* + ‘Richmond pliosaur’ (QM F18041) + *Sulcusuchus* clade.

The *a posteriori* method to identify wildcard taxa found the following OTUs to be unstable and they were therefore removed from the pruned dataset: *Pistosaurus* (skull), *H. valdensis*, *Eromangasaurus australis*, *Tuarangisaurus keyesi*, *Pliosaurus irgisensis*, *Pliosaurus brachyspondylus*, *Pliosaurus rossicus*, *Gallardosaurus iturraldei*, QMF 51291, *Polyptychodon* sp. and *Georgiasaurus penzensis*. *Thililua longicollis* was also regarded as a wildcard taxon, but was retained in the reduced consensus analysis as it is a focus of the current work (yet the position of *Thililua longicollis* and its closely related taxa is fully resolved in the results of the pruned dataset; the removal of *Manemergus anguirostris* is sufficient to recover well-resolved tree for Polycotylidae). Initial analysis of the pruned dataset recovered 2528 most parsimonious trees with a length of 1678 steps, with a strict consensus topology similar to that of our pruned dataset (see below), but with *Edgarosaurus* and *Manemergus* as the earliest-branching polycotylids. We suspected that the finding of the holotype of *Manemergus* in this basal position resulted from its highly juvenile ontogenetic status, and that this was influencing the optimization of character states in polycotylid phylogeny. Therefore, we conducted the analysis excluding *Manemergus*, in addition to the wildcard taxa listed above. This yielded 3584 most parsimonious trees each with a length of 1667 steps (figure 8; electronic supplementary material, figure S3). The rescaled consistency index of these trees is 0.1662. The trees have a very good fit to stratigraphy (electronic supplementary material, ESM1 and figure S4). The strict consensus of analysis of this reduced dataset yielded a well-resolved Leptocleidia. *Edgarosaurus muddi* is recovered as the earliest-branching polycotylid. No clade in our reduced consensus could bear the name Palmulasaurinae as both *Pahasapasaurus* and *Palmulasaurus* form an unresolved polytomy as sister taxa to the more derived polycotylids. This polytomy could be resolved by information from future studies or new discoveries as either a clade (to which ‘Palmulasaurinae’ could be correctly applied) or a paraphyletic grade.

*Plesiopleurodon* and the ‘Richmond pliosaur’ (QM F18041) form a clade that we also find to include the Maastrichtian taxon *Sulcusuchus erraini*. We hereby chose to formally name and define this clade, Occultonectia clade nov. (see below). *Thililua longicollis* and *Mauriciosaurus fernandezi* form the successive sister lineages of Polycotylinae. The generic content of Polycotylinae is similar to that specified by Albright *et al.* [15]: *Eopolycotylus*, *Polycotylus*, *Trinacromerum* and *Dolichorhynchops*. In both datasets, we failed to recover a monophyletic *Dolichorhynchops*, notably because *Dolichorhynchops tropicensis* occupies a more basal position among polycotylines and because *Georgiasaurus penzensis* forms an unresolved clade with the species *Dolichorhynchops herschelensis*, *Dolichorhynchops bonneri* and *Dolichorhynchops* sp. (ROM 29010) (in the analysis of the full dataset). This questions the generic attribution of *Dolichorhynchops tropicensis.*

### 5.2. Tempo of polycotylid evolution

Uncertainty has surrounded the timing and intensity of the early polycotylid radiation. Indeed, the presence of derived leptocleidids during the Valanginian (*Leptocleidus capensis*) results in a lengthy basal polycotylid ghost lineage that extends from the earliest Cretaceous up to the earliest fossil records of polycotylids, the ‘Richmond pliosaur’ (QM F18041) from the Upper Albian of Australia [67] and *Edgarosaurus muddi* from the Upper Albian of North America [14]. The phylogenetic position of *Plesiopleurodon* implies that a series of cladogenetic events took place before the Early–Late Cretaceous boundary: at least seven polycotylid lineages (including the ancestor of polycotylines) were present by the end of the Middle Albian (figure 8) and our phylogenetic diversity estimates suggest that this number possibly reached 11 (see electronic supplementary material, table S4 for the phylogenetic diversity estimates, under different methods of reconstruction of branch lengths and datasets).

The Cenomanian stage probably represents the most important turnover in the evolution of polycotylids, witnessing both the sudden radiation of all or nearly all polycotylines lineages (at least eight lineages by the Early Turonian) and the extinction of many non-polycotylines, with the exception of the occultonectian polycotylids, which are represented by *Sulcusuchus* in the Late Campanian or Early Maastrichtian.

### 5.3. Phylogenetic definitions

As discussed by O'Keefe [16], the definition of Palmulasaurinae is difficult to use, as it has been solely based on *Palmulasaurus quadratus* and UMUT MV 19965, a poorly known specimen from Japan. The content of the inclusive clade containing *Palmulasaurus* and excluding polycotylines and *Edgarosaurus* (as per Albright *et al*. [15]) has been widely varying in most phylogenetic analyses of polycotylids, including our full and reduced datasets. Therefore, we concur with O'Keefe [16] that the phylogenetic data presently available prevent the use and the formalization of the clade Palmulasaurinae. A stable clade of non-polycotyline polycotylids unites *Plesiopleurodon wellesi* and the ‘Richmond pliosaur’ (QM F18041)—this association has been recovered in several other studies [9,19,27,34,68], and we find for the first time that it also includes *Sulcusuchus erraini*. We formally erect the clade Occultonectia clade nov. for reception of these species.

Occultonectia clade nov.

*Etymology*. From the Latin *occultus*, meaning hidden, concealed, and the Latinized Greek *nectes (νήκτης)* meaning swimmer. This refers to the fact that all the members of Occultonectia have been interpreted as non-polycotylids in the past (as crocodyliforms, pliosaurids and leptocleidians). Furthermore, a long ghost lineage unites the Late Campanian or Early Maastrichtian occultonectian *Sulcusuchus erraini* with other members of the clade (figure 8). *Sulcusuchus erraini* is found in freshwater-dominated estuarine deposits of the La Colonia Formation [69] and exhibits small body size [31] consistent with its occurrence in a restricted environment. We therefore propose the survival of occultonectians, ‘concealed’ in freshwater or marginal marine refugia up until the final extinction of polycotylids.

*Phylogenetic definition*. The branch-based clade comprising all taxa more closely related to *Plesiopleurodon wellesi* than to *Polycotylus latipinnis*, *Pahasapasaurus haasi* or *Palmulasaurus quadratus*. Occultonectia should not be used in cases where the specifiers of Palmulasaurinae (*Palmulasaurus quadratus*) or Polycotylinae (*Polycotylus latipinnis*) are found to be more closely related to *Sulcusuchus erraini* than to *Plesiopleurodon wellesi* or the ‘Richmond plesiosaur’.

*Diagnosis*. Occultonectia is supported by the following non-unique unambiguous synapomorphies in the analysis of the full dataset: (i) reduced preorbital skull length (in between 0.45 and 0.55 compared with the total skull length) (4.2 → 4.1); (ii) undulating, ‘scalloped’ alveolar margin of the upper jaw in lateral view (13.0 → 13.1); (iii) elongate dorsomedian ridge of the premaxilla, extending from the interorbital region to the tip of the rostrum (19.1 → 19.2); (iv) raised ventral keel on the symphysis (114.0 → 114.1); (v) coarse longitudinal enamel ridges (137.1 → 137.0); (vi) large number of maxillary teeth (greater than 28) (138.1 → 138.2); (vii) absence of a second postaxial accessory ossicle articulating with propodial (233.1 → 233.0) and (viii) narrow forefin (aspect ratio in between 3.1 and 3.5 (235.0 → 235.1)).

The analysis of the pruned dataset recovered 12 non-unique unambiguous synapomorphies supporting Occultonectia: those found in the full dataset (except iv, v and viii) plus the following ones: rounded dorsal crest of the squamosal in the squamosal arch (54.1 → 54.0); anteroposteriorly short posterior interpterygoid vacuities (103.2 → 103.0); long angular that does participate to the mandibular symphysis (126.2 → 126.1); elongated dorsoventral groove on the posterior surface of posterior cervical and dorsal neural spines (170.0 → 170.1); (ix) few dorsal vertebra (17–19) (179.1 → 179.0); (x) absence of foramina or perforations on the coracoid (211.1 → 211.0) and (xi) sigmoidal ilium (221.1 → 221.2).

### 5.4. Pattern of morphospace occupation

**Figure 9.**
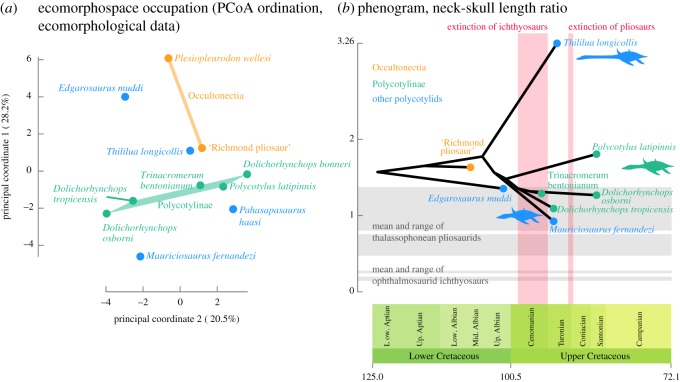
Morphospace occupation and evolution of the neck of polycotylid plesiosaurians. (*a*) Morphospace occupation of polycotylids visualized using the first two principal coordinates of the PCoA ordination. Occultonectia and Polycotylinae occupy clearly distinct regions of the morphospace. (*b*) Evolution of neck proportions of polycotylids, with maximum-likelihood reconstruction of ancestral states. Silhouettes represent the gross differences in body proportions. The phenogram is superimposed on the range and mean of neck/skull length ratios of thalassophonean pliosaurids [4,9] and ophthalmosaurid ichthyosaurs [70] and the extinction windows of these clades.

We visualized the morphospace of polycotylids by applying principal coordinate analyses to the Bauplan dataset that summarizes the body shape of polycotylids (figure 9; electronic supplementary material, ESM1, ESM7 and tables S1–S3). We also computed a phylomorphospace, visible in electronic supplementary material, ESM1 and figure S5. The earliest polycotylid *Edgarosaurus muddi* and the occultonectian *Plesiopleurodon* occupy a distinct region of the morphospace, especially in the first principal coordinate. This morphotype is convergent with that of pliosaurids [9]. Despite its phylogenetic position, which is intermediate between *Edgarosaurus* and Occultonectia (figure 8), *Pahasapasaurus haasi* falls close to a region of the morphospace that is otherwise typical of the derived, polycotyline polycotylids such as *Dolichorhynchops*, predominantly due to the presence of a long, gracile snout and dentition. By contrast, the successive sister taxa of polycotylines, *Thililua longicollis* and *Mauriciosaurus fernandezi* occupy diametrically opposed regions of the morphospace, somewhat surrounding the polycotyline region but clearly outside it. Despite their high taxic diversity during the Late Cretaceous, and especially the Campanian (figure 8), polycotylines occupy a very restricted region of the polycotylid morphospace (at least in the two principal coordinates), where the intrageneric variability (i.e. that of *Dolichorhynchops*) appears at least equally as large as the intergeneric variability, especially in the second principal coordinate.

### 5.5. Disparity over time

Polycotylid disparity only weakly fluctuates over time (figure 8; electronic supplementary material, table S5). The Albian assemblage contains *Edgarosaurus* and the occultonectian ‘Richmond pliosaur’ (QM F18041), yielding a sum of variances of 10.97. Disparity augments and peaks during the Cenomanian, thanks to the sampled co-occurrence of occultonectians (*Plesiopleurodon*), early polycotylids (*Pahasapasaurus*) and polycotylines (*Trinacromerum*), with a value of 12.8. The absence of analysable occultonectians in subsequent time bins (electronic supplementary material, tables S1–S3) results in an apparent drop of disparity (sum of range = 9.26), despite the co-occurrence of wildly varying taxa in the Turonian such as *Thililua*, *Mauriciosaurus*, *Palmulasaurus* and polycotylines. It is likely that the Turonian will record the highest polycotylid disparity once a Turonian occultonectian is discovered. Nevertheless, the Campanian–Maastrichtian bin only samples species within the genus *Dolichorhynchops* and yields a sum of range that is just slightly inferior to the value obtained for the Turonian (9.21 versus 9.26), suggesting quite an amount of morphological variability within this speciose genus.

### 5.6. Evolution of relative neck size

Without *Thililua longicollis* and *Mauriciosaurus fernandezi*, the evolution of the neck/skull length ratio of polycotylid would be trivial: our maximum-likelihood reconstruction of ancestral states suggests a neck/skull length ratio approximately 1.70 for the polycotylid ancestor, and most polycotylids deviate only weakly from this value: *Dolichorhynchops tropicensis* has a neck that is roughly as long as its skull (ratio = 1.09), while *Polycotylus latipinnis* possesses a skull that is slightly less than twice as long as its skull length (ratio = 1.81). All other polycotylids, except *Thililua longicollis* and *Mauriciosaurus fernandezi*, are constrained within this range. Polycotylines show just a slightly more expanded range (from 1.09 to 1.81) than early polycotylids (*Edgarosaurus muddi* and the ‘Richmond pliosaur’ (QM F18041), from 1.35 to 1.63).

*Mauriciosaurus fernandezi* and *Thililua longicollis* markedly expand the range of polycotylid relative neck sizes: *Mauriciosaurus* is the sole polycotylid that possesses a neck that is shorter than its skull length, with a value that is close to the mean ratio of pliosaurids (figure 9). On the other hand, *Thililua longicollis* has a neck that is 3.26 times the length of its skull. Among plesiosaurians, the cryptoclidids *Cryptoclidus eurymerus* (3.29; L.S. Soul 2017, personal communication) and *Tricleidus seeleyi* (3.32; [71]; L.S. Soul 2017, personal communication) and the early plesiosaurian *Attenborosaurus conybeari* (3.48; [72]; L.S. Soul 2017, personal communication) possess comparable values. However, in cryptoclidids, the high ratio results predominantly from the presence of a small, shortened skull and is therefore quite distinct from the presence of a long neck and large skull with proportionally elongated rostrum in *Thililua*.

Polycotylids are known to have evolved convergently with pliosaurids [1,6,9] and derived polycotylids have been regarded as fast predators, bearing some similarities with thunnosaurian ichthyosaurs [73,74]. Both pliosaurids and ichthyosaurs disappeared over the course of the Cenomanian–Turonian interval [70,75,76]; it is thus possible that derived polycotylids might have evolved to occupy some of these vacated ecological niches. We computed the mean and global range of neck–skull length ratio as a proxy to investigate the evolution of the gross body shape of polycotylids and detect changes that might be related to the extinction of pliosaurids and ichthyosaurs. As expected, polycotylids never evolved a neck/skull length ratio remotely close to that of ophthalmosaurid ichthyosaurs, either before or after the extinction of ichthyosaurs. Thalassophonean pliosaurids also have, in general, shorter necks than polycotylids, although *Edgarosaurus muddi*, *Mauriciosaurus fernandezi* and polycotylines (with the exception of *Polycotylus latipinnis*, which has a longer neck) enter the range of thalassophonean pliosaurids. *Mauriciosaurus fernandezi* is the sole polycotylid to have a relative neck size that is roughly similar to that of thalassophoneans. Our time-scaled phenogram with maximum-likelihood reconstruction of ancestral states suggests that *Edgarosaurus muddi*, *Mauriciosaurus fernandezi* and the polycotyline ancestor evolved thalassophonean-like neck lengths prior to the extinction of Pliosauridae in the Turonian.

## 6. Discussion

### 6.1. The evolutionary radiation of polycotylids

Polycotylids are the youngest family of plesiosaurians, entering the fossil record during the Albian. Nevertheless, occurrences of their sister taxon Leptocleididae indicate a long ghost lineage for the origin of Polycotylidae extending back to the Berriasian. ‘Equal’ reconstruction of branch lengths (see Brusatte *et al*. [42]) thus creates a gradual and somewhat artificial increase of the phylogenetic diversity over the Berriasian–Albian interval (figure 8). New early polycotylids and sister taxa to Polycotylidae are needed to better reconstruct the evolution of their diversity during the Early Cretaceous; scant fossil evidence [77,78] and phylogenetic topology (with the Albian occurrence of the occultonectian ‘Richmond pliosaur’ (QM F18041)) suggest that we are still missing the earliest representatives of this clade.

Polycotylids (figure 8) are unusual among other fast-swimming marine reptiles of the Early Cretaceous in increasing their diversity and their disparity during the Late Albian–Turonian interval; ichthyosaurs met their demise in a two-step extinction event during and at the end of the Cenomanian [70,79], while the last pliosaurids had low disparity, belonging to the same global morphotype [9], and disappeared by the Middle Turonian [76]. The phylogenetic diversity estimate of polycotylids seems to follow the global temperature trend, decreasing after the Turonian climatic optimum. To test this, we used the median phylogenetic diversity estimate, using equal reconstruction of branch lengths of the most parsimonious trees from the analysis of the pruned dataset (figure 8*c*; electronic supplementary material, table S4) and compared it to the isotopic record reported in Prokoph *et al*. [80], binned per stage (sub-stages for the Aptian: Lower and Upper and for the Albian: Lower, Middle, Upper). The phylogenetic diversity of polycotylids is negatively correlated to the mean ∂18O (thus indicating a positive correlation with temperature), but this correlation is not significant at *α* = 0.05: Pearson's *r* = −0.46, *p*-value = 0.07964. A comparable situation is found when comparing the phylogenetic diversity estimates with the volatility of temperatures (computed as the variance of ∂18O per bin; see [70]): Pearson’s *r* = 0.499, *p*-value = 0.05782. The maximal disparity of polycotylids is found during the Cenomanian, thanks to the co-occurrence of polycotylines (with *Trinacromerum bentonianum*) and the thalassophonean-like occultonectian *Plesiopleurodon wellesi*. The absence of sampled occultonectians in the Turonian results in an apparent drop of disparity, despite the co-occurrence of taxa that are widely distinct in general body shape: *Thililua longicollis* and *Mauriciosaurus fernandezi* (figure 9).

### 6.2. The peculiar morphology of *Thililua*

*Thililua longicollis* combines an elongated skull with a long neck and teeth-bearing carinae. These features yield discordant suggestions for its possible ecology. Indeed, an elongated skull is often associated with fast swimming [74], which is poorly compatible with such an elongated neck [81,82]. Similarly, the co-occurrence of an elongated neck and short, carinated, dorsoventrally oriented teeth has never been observed in plesiosaurians [66]. Among polycotylids, both the long neck size and the carinated teeth are only recorded in *Thililua longicollis*, suggesting that these features are not plesiomorphic retentions. This combination of features does not match with the purported mesoscopic prey filterer niche suggested by Noè *et al*. [66] for long-necked plesiosaurians and implies a unique yet still mysterious ecology for *Thililua longicollis*.

### 6.3. Is the diversification of polycotylids an ecological release?

Our analyses (figure 9) suggest that polycotylids repeatedly evolved a thalassophonean-like neck–skull length ratio. The first polycotylids to enter the fossil record (*Plesiopleurodon wellesi* and *Edgarosaurus muddi*) are the most pliosaurid-like. Nevertheless, our phylogeny suggests a more complex evolutionary history, as *Pahasapasaurus haasi* (which is more derived than *Edgarosaurus muddi*, but less so than occultonectians) occupies a morphological position similar to that of polycotylines, with a relative snout length, a relative symphysis length and a number of teeth that are quite distinct from those of thalassophonean-like polycotylids. Polycotylids evolved thalassophonean-like relative neck lengths numerous times and always before the extinction of pliosaurids. Thus, our results suggest that polycotylids did not radiate to fill the morphospace vacated by the extinctions of ichthyosaurs and pliosaurids, but achieved these general skull/neck proportions earlier. Our analyses are, however, limited in taxonomic scope and could not detect craniodental convergences between these clades. Using craniodental and postcranial data, Fischer *et al*. [9] showed that pliosaurids were mostly similar to *Edgarosaurus muddi* and the occultonectian *Plesiopleurodon wellesi*, whereas polycotylines occupy a distinct region of the morphospace; when coeval, thalassophoneans and polycotylines thus probably occupied distinct ecological niches.

The diversification of polycotylids during the earliest Late Cretaceous does not seem to have been permitted by the extinction of ichthyosaurs and pliosaurids, because most polycotylid morphotypes evolved prior to the extinctions of these clades. Nevertheless, the Cenomanian–Turonian transition marks a profound turnover in the evolutionary history of polycotylids, with extinction of key early diverging lineages (except *Palmulasaurus quadratus*, which probably disappeared slightly after, during the Turonian, and the lineage leading to the Campanian–Maastrichtian occultonectian *Sulcusuchus erraini*) and the abrupt rise to dominance of polycotylines and closely related taxa. The co-occurrence of *Thililua longicollis*, *Mauriciosaurus fernandezi* and the earliest polycotylines during the Turonian suggests that these polycotylids colonized a series of different niches, but none that are similar to mid-Cretaceous thalassophonean pliosaurids. We posit that the Cenomanian–Turonian diversification and turnover of polycotylids might be better explained by the global turnover and changes in the marine realm that occurred during the early Late Cretaceous [70,75,76,83] than a consequence of the extinction of their possible competitors.

On a broad scale, it is possible that the dynamics of marine vertebrate clades through the Cenomanian–Turonian interval represent clade-specific responses to environmental upheavals such as the Early Turonian thermal maximum. Polycotylids and early mosasauroids (e.g. [84]) increased in abundance, taxic richness and morphological disparity during that time interval. Ichthyosaurs and pliosaurids, which had long preceding intervals of persistence in marine ecosystems, became extinct. Animals occupying lower tiers of marine trophic webs also exhibit a wide range of responses to these events: some groups suffered from intense, staggered extinctions such as ammonites [85–87], while others went through a profound turnover, such as in ‘reef’-making, hippuritoid bivalves [88–90], or show an intense but brief radiation, such as acanthomorph actinopterygians and multiple elasmobranch clades [91–95]. These divergent responses to environmental change ultimately restructured the composition of marine ecosystems of the final intervals of the Cretaceous, but the causes of the disappearance of polycotylids from the fossil record prior to the end of the Maastrichtian remain unclear.

## 7. Conclusion

— *Thililua longicollis* does not have a highly unusual cranial architecture, but nevertheless possesses a series of distinct traits. Among these, the combination of long neck, long skull and carinated teeth is unique among Plesiosauria.
— *Manemergus* may be congeneric with *Thililua*, but the very young age of the type and only known specimen of *M. anguirostris* makes it difficult to reach a taxonomic decision.
— *Plesiopleurodon wellesi*, *S. erraini* and the ‘Richmond pliosaur’ (QM F18041) form a new clade of early branching polycotylids, Occultonectia clade nov., with a Bauplan that is clearly distinct for that of polycotyline polycotylids.
— Polycotylids reach their maximal disparity during the Cenomanian with co-occurrence of occultonectians and the earliest polycotylines.
— Volatility in neck size and general body shape is most evident during the Cenomanian–Turonian interval, before the fixation of the constrained polycotyline Bauplan. Short necks did not evolve as a consequence of an ecological release following the extinction of ichthyosaurs and pliosaurids.
— The evolution of Polycotylidae during the Cenomanian–Turonian interval contrasts with patterns seen in other large pelagic marine reptiles and suggests different responses to the global changes.

## Supplementary Material

Supplementary Information

## Supplementary Material

Full Phylogenetic dataset

## Supplementary Material

Taxon ages for full phylogenetic dataset

## Supplementary Material

Pruned Phylogenetic dataset

## Supplementary Material

Taxon ages for pruned phylogenetic dataset

## Supplementary Material

Numerical time bins

## Supplementary Material

Bauplan/morphological data

## Supplementary Material

R script

## Data accessibility

Our data, as well as the R script written to generate results and figures, are deposited at Dryad: http://dx.doi.org/10.5061/dryad.4gh8b [96].

## References

[1] O'Keefe FR 2002 The evolution of plesiosaur and pliosaur morphotypes in the Plesiosauria (Reptilia: Sauropterygia). Palaeobiology 28, 101–112. (doi:10.1666/0094-8373(2002)028<0101:TEOPAP>2.0.CO;2)

[2] O'Keefe FR, Carrano MT 2005 Correlated trends in the evolution of the plesiosaur locomotor system. Palaeobiology 31, 656–675. (doi:10.1666/04021.1)

[3] Benson RBJ, Druckenmiller PS 2014 Faunal turnover of marine tetrapods during the Jurassic–Cretaceous transition. Biol. Rev. 89, 1–23. (doi:10.1111/brv.12038) 23581455 10.1111/brv.12038

[4] Soul LC, Benson RBJ 2017 Developmental mechanisms of macroevolutionary change in the tetrapod axis: a case study of Sauropterygia. Evolution 71, 1164–1177. (doi:10.1111/evo.13217) 28240769 10.1111/evo.13217 PMC5485078

[5] Williston SW 1903 North American plesiosaurs. Part I. F. Colomb. Museum Publ. Geol. Ser. 2, 1–206. (doi:10.5962/bhl.title.3497)

[6] O'Keefe FR 2001 A cladistic analysis and taxonomic revision of the Plesiosauria (Reptilia: Sauropterygia). Acta Zool. Fenn. 213, 1–63.

[7] Ketchum HF, Benson RBJ 2010 Global interrelationships of Plesiosauria (Reptilia, Sauropterygia) and the pivotal role of taxon sampling in determining the outcome of phylogenetic analyses. Biol. Rev. 85, 361–392. (doi:10.1111/j.1469-185X.2009.00107.x) 20002391 10.1111/j.1469-185X.2009.00107.x

[8] O'Keefe RF 2004 Preliminary description and phylogenetic position of a new plesiosaur (Reptilia: Sauropterygia) from the Toarcian of Holzmaden, Germany. J. Paleontol. 78, 973–988. (doi:10.1666/0022-3360(2004)078<0973:PDAPPO>2.0.CO;2)

[9] Fischer V, Benson RBJ, Zverkov NG, Soul LC, Arkhangelsky MS, Lambert O, Stenshin IM, Uspensky GN, Druckenmiller PS 2017 Plasticity and convergence in the evolution of short-necked plesiosaurs. Curr. Biol. 27, 1667–1676. (doi:10.1016/j.cub.2017.04.052) 28552354 10.1016/j.cub.2017.04.052

[10] Smith AS, Dyke GJ 2008 The skull of the giant predatory pliosaur *Rhomaleosaurus cramptoni*: implications for plesiosaur phylogenetics. Naturwissenschaften 95, 975–980. (doi:10.1007/s00114-008-0402-z) 18523747 10.1007/s00114-008-0402-z

[11] Druckenmiller PS, Russell AP 2009 A phylogeny of Plesiosauria (Sauropterygia) and its bearing on the systematic status of *Leptocleidus* Andrews, 1922. Zootaxa 1863, 1–120.

[12] Kear BP 2005 Marine reptiles from the Lower Cretaceous (Aptian) deposits of White Cliffs, southeastern Australia: implications of a high latitude, cold water assemblage. Cretac. Res. 26, 769–782. (doi:10.1016/j.cretres.2005.04.006)

[13] Druckenmiller PS, Russell AP 2009 Earliest North American occurrence of Polycotylidae (Sauropterygia: Plesiosauria) from the Lower Cretaceous (Albian) Clearwater formation, Alberta, Canada. J. Paleontol. 83, 981–989. (doi:10.1666/09-014.1)

[14] Druckenmiller PS 2002 Osteology of a new plesiosaur from the Lower Cretaceous (Albian) Thermopolis Shale of Montana. J. Vertebr. Paleontol. 22, 29–42. (doi:10.1671/0272-4634(2002)022[0029:OOANPF]2.0.CO;2)

[15] Albright LB, Gillette DD, Titus AL 2007 Plesiosaurs from the Upper Cretaceous (Cenomanian–Turonian) Tropic Shale of southern Utah, part 2: Polycotylidae. J. Vertebr. Paleontol. 27, 41–58. (doi:10.1671/0272-4634(2007)27[41:PFTUCC]2.0.CO;2)

[16] O'Keefe RF 2008 Cranial anatomy and taxonomy of *Dolichorhynchops bonneri* new combination, a polycotylid (Sauropterygia: Plesiosauria) from the Pierre Shale of Wyoming and South Dakota. J. Vertebr. Paleontol. 28, 664–676. (doi:10.1671/0272-4634(2008)28[664:CAATOD]2.0.CO;2)

[17] O'Keefe FR, Street HP 2009 Osteology of the cryptocleidoid plesiosaur *Tatenectes laramiensis*, with comments on the taxonomic status of the Cimoliasauridae. J. Vertebr. Paleontol. 29, 48–57. (doi:10.1671/039.029.0118)

[18] Otero RA 2016 Taxonomic reassessment of *Hydralmosaurus* as *Styxosaurus*: new insights on the elasmosaurid neck evolution throughout the Cretaceous. PeerJ 4, e1777 (doi:10.7717/peerj.1777) 27019781 10.7717/peerj.1777 PMC4806632

[19] Schumacher BA, Martin JE 2015 *Polycotylus latipinnis* Cope (Plesiosauria, Polycotylidae), a nearly complete skeleton from the Niobrara Formation (Early Campanian) of southwestern South Dakota. J. Vertebr. Paleontol. 36, e1031341 (doi:10.1080/02724634.2015.1031341)

[20] Bardet N, Pereda Suberbiola X, Jalil N-E 2003 A new polycotylid plesiosaur from the Late Cretaceous (Turonian) of Morocco. C. R. Palevol. 2, 307–315. (doi:10.1016/S1631-0683(03)00063-0)

[21] Welles SP 1943 Elasmosaurid plesiosaurs with description of new material from California and Colorado. Mem. Univ. Calif. 13, 125–254.

[22] Sato T 2002 Description of plesiosaurs (Reptilia: Sauropterygia) from the Bearpaw Formation (Campanian–Maastrichtian) and a phylogenetic analysis of the Elasmosauridae. PhD thesis, University of Calgary.

[23] Wiffen J, Moisley WL 1986 Late Cretaceous reptiles (Families Elasmosauridae and Pliosauridae) from the Mangahouanga Stream, North Island, New Zealand. New Zeal. J. Geol. Geophys. 29, 205–252. (doi:10.1080/00288306.1986.10427535)

[24] Martin JE, Buffetaut E, Naksri W, Lauprasert K, Claude J 2012 *Gavialis* from the Pleistocene of Thailand and its relevance for drainage connections from India to Java. PLoS ONE 7, e44541 (doi:10.1371/journal.pone.0044541) 23028557 10.1371/journal.pone.0044541 PMC3445548

[25] Buchy M-C, Métayer F, Frey E 2005 Osteology of *Manemergus anguirostris* n. gen. et sp., a new plesiosaur (Reptilia, Sauropterygia) from the Upper Cretaceous of Morocco. Palaeontographica 272, 97–120.

[26] Druckenmiller PS, Knutsen EM 2012 Phylogenetic relationships of Upper Jurassic (Middle Volgian) plesiosaurians (Reptilia: Sauropterygia) from the Agardhfjellet Formation of central Spitsbergen, Norway. Nor. J. Geol. 92, 277–284.

[27] Benson RBJ, Ketchum HF, Naish D, Turner LE 2013 A new leptocleidid (Sauropterygia, Plesiosauria) from the Vectis Formation (early Barremian–early Aptian; Early Cretaceous) of the Isle of Wight and the evolution of Leptocleididae, a controversial clade. J. Syst. Palaeontol. 11, 231–248. (doi:10.1080/14772019.2011.634444)

[28] Carpenter K 1996 A review of short-necked plesiosaurs from the Cretaceous of the Western Interior, North America. Neues Jahrb. Geol. Paläontol. Abh. 201, 259–287.

[29] Kear BP, Barrett PM 2011 Reassessment of the Lower Cretaceous (Barremian) pliosauroid *Leptocleidus superstes* Andrews, 1922 and other plesiosaur remains from the nonmarine Wealden succession of southern England. Zool. J. Linn. Soc. 161, 663–691. (doi:10.1111/j.1096-3642.2010.00648.x)

[30] McKean R 2012 A new species of polycotylid plesiosaur (Reptilia: Sauropterygia) from the Lower Turonian of Utah: extending the stratigraphic range of *Dolichorhynchops*. Cretac. Res. 34, 184–199. (doi:10.1016/j.cretres.2011.10.017)

[31] O'Gorman JP, Gasparini Z 2013 Revision of *Sulcusuchus erraini* (Sauropterygia, Polycotylidae) from the Upper Cretaceous of Patagonia, Argentina. Alcheringa Aust. J. Palaeontol. 37, 163–176. (doi:10.1080/03115518.2013.736788)

[32] Gasparini ZB, Spalletti LA 1990 Un nuevo cocodrilo en depósitos mareales maastrichtianos de la Patagonia noroccidental. Ameghiniana 27, 141–150.

[33] Frey E, Mulder EWA, Stinnesbeck W, Rivera-Sylva HE, Padilla-Gutiérrez JM, González-González AH 2017 A new polycotylid plesiosaur with extensive soft tissue preservation from the early Late Cretaceous of northeast Mexico. Bol. Soc. Geol. Mex. 69, 87–134. (doi:10.18268/BSGM2017v69n1a5)

[34] Fischer V, Arkhangelsky MS, Stenshin IM, Uspensky GN, Zverkov NG, Benson RBJ 2015 Peculiar macrophagous adaptations in a new Cretaceous pliosaurid. R. Soc. open sci. 2, 150552 (doi:10.1098/rsos.150552) 27019740 10.1098/rsos.150552 PMC4807462

[35] Serratos DJ, Druckenmiller P, Benson RBJ 2017 A new elasmosaurid (Sauropterygia, Plesiosauria) from the Bearpaw Shale (Late Cretaceous, Maastrichtian) of Montana demonstrates multiple evolutionary reductions of neck length within Elasmosauridae. J. Vertebr. Paleontol. 1278608, 1–25. (doi:10.1080/02724634.2017.1278608)

[36] Ochev VG 1976 A new pliosaur from the Upper Cretaceous of Penza Province. Paleontol. J. 2, 135–138.

[37] Sato T, Wu X-C, Tirabasso A, Bloskie P 2011 Braincase of a polycotylid plesiosaur (Reptilia: Sauropterygia) from the Upper Cretaceous of Manitoba, Canada. J. Vertebr. Paleontol. 31, 313–329. (doi:10.1080/02724634.2011.550358)

[38] Sato T 2005 A new polycotylid plesiosaur (Reptilia: Sauropterygia) from the Upper Cretaceous Bearpaw Formation in Saskatchewan, Canada. J. Paleontol. 79, 969–980. (doi:10.1666/0022-3360(2005)079[0969:ANPPRS]2.0.CO;2)

[39] Goloboff PA, Catalano SA 2016 TNT version 1.5, including a full implementation of phylogenetic morphometrics. Cladistics 32, 276–296. (doi:10.1111/cla.12160) 10.1111/cla.12160 34727670

[40] Pol D, Escapa IH 2009 Unstable taxa in cladistic analysis: identification and the assessment of relevant characters. Cladistics 25, 515–527. (doi:10.1111/j.1096-0031.2009.00258.x) 10.1111/j.1096-0031.2009.00258.x 34879625

[41] Paradis E, Claude J, Strimmer K 2004 APE: Analyses of phylogenetics and evolution in R language. Bioinformatics 20, 289–290. (doi:10.1093/bioinformatics/btg412) 14734327 10.1093/bioinformatics/btg412

[42] Brusatte SL, Benton MJ, Ruta M, Lloyd GT 2008 Superiority, competition, and opportunism in the evolutionary radiation of dinosaurs. Science 321, 1485–1488. (doi:10.1126/science.1161833) 18787166 10.1126/science.1161833

[43] Bapst DW 2012 paleotree: an R package for paleontological and phylogenetic analyses of evolution. Methods Ecol. Evol. 3, 803–807. (doi:10.1111/j.2041-210X.2012.00223.x)

[44] R Core Team. 2016 R: A language and environment for statistical computing. Vienna, Austria: R Foundation for Statistical Computing. See https://www.R-project.org/.

[45] Norell MA 1992 Taxic origin and temporal diversity: the effect of phylogeny. In Extinction and phylogeny (eds Novacek MJ, Wheeler QD), pp. 89–118. New York, NY: Columbia University Press.

[46] Signor PW, Lipps JH 1982 Sampling bias, gradual extinction patterns and catastrophes in the fossil record. Geol. Soc. Am. Spec. Pap. 190, 291–296. (doi:10.1130/SPE190-p291)

[47] Wagner PJ 2000 The quality of the fossil record and the accuracy of phylogenetic inferences about sampling and diversity. Syst. Biol. 49, 65–86. (doi:10.1666/0094-8373(2000)26[341:PAATFR]2.0.CO;2) 12116484 10.1080/10635150050207393

[48] Bell MA, Lloyd GT 2015 strap: an R package for plotting phylogenies against stratigraphy and assessing their stratigraphic congruence. Palaeontology 58, 379–389. (doi:10.1111/pala.12142)

[49] Zeileis A, Grothendieck G 2005 Zoo: S3 infrastructure for regular and irregular time series. J. Stat. Softw. 14, 1–27. (doi:10.18637/jss.v014.i06)

[50] Revell LJ 2012 phytools: an R package for phylogenetic comparative biology (and other things). Methods Ecol. Evol. 3, 217–223. (doi:10.1111/j.2041-210X.2011.00169.x)

[51] Knutsen EM, Druckenmiller PS, Hurum JH 2012 Two new species of long-necked plesiosaurians (Reptilia: Sauropterygia) from the Upper Jurassic (Middle Volgian) Agardhfjellet Formation of central Spitsbergen. Nor. J. Geol. 92, 187–212.

[52] White TE 1940 Holotype of *Plesiosaurus longirostris* Blake and classification of the plesiosaurs. J. Paleontol. 14, 451–467.

[53] Benson RBJ, Ketchum HF, Noè LF, Gómez-Pérez M 2011 New information on *Hauffiosaurus* (Reptilia, Plesiosauria) based on a new species from the Alum Shale member (Lower Toarcian: Lower Jurassic) of Yorkshire, UK. Palaeontology 54, 547–571. (doi:10.1111/j.1475-4983.2011.01044.x)

[54] Ferrandini M, Philip J, Babinot J-F, Ferrandini J, Tronchetti G 1985 La plate-forme carbonatée du Cénomano-Turonien de la région d'Erfoud-Errachidia (Sud-Est marocain): stratigraphie et paléoenvironnements. Bull. Soc. Géol. Fr. 8, 559–564.

[55] Lebedel V, Lézin C, Andreu B, Ettachfini EM, Grosheny D 2015 The upper Cenomanian-lower Turonian of the Preafrican Trough (Morocco): platform configuration and palaeoenvironmental conditions. J. Afr. Earth Sci. 106, 1–16. (doi:10.1016/j.jafrearsci.2015.03.001)

[56] Schumacher BA 2007 A new polycotylid plesiosaur (Reptilia; Sauropterygia) from the Greenhorn Limestone (Upper Cretaceous; lower upper Cenomanian), Black Hills, South Dakota. Geol. Soc. Am. Spec. Pap. 427, 133–146. (doi:10.1130/2007.2427(09))

[57] Benson RBJ, Evans M, Smith AS, Sassoon J, Moore-Faye S, Ketchum HF, Forrest R 2013 A giant pliosaurid skull from the Late Jurassic of England. PLoS ONE 8, e65989 (doi:10.1371/journal.pone.0065989) 23741520 10.1371/journal.pone.0065989 PMC3669260

[58] McHenry CR 2009 ‘Devourer of Gods’. The palaeoecology of the Cretaceous pliosaur *Kronosaurus queenslandicus*. PhD thesis, University of Newcastle, Australia.

[59] Welles SP 1952 A review of the North American Cretaceous elasmosaurs. Univ. Calif. Publ. Geol. Sci. 29, 47–144.

[60] O'Keefe RF 2004 On the cranial anatomy of the polycotylid plesiosaurs, including new material of *Polycotylus latipinnis*, Cope, from Alabama. J. Vertebr. Paleontol. 24, 326–340. (doi:10.1671/1944)

[61] Ketchum HF, Benson RBJ 2011 The cranial anatomy and taxonomy of *Peloneustes philarchus* (Sauropterygia, Pliosauridae) from the Peterborough Member (Callovian, Middle Jurassic) of the United Kingdom. Palaeontology 54, 639–665. (doi:10.1111/j.1475-4983.2011.01050.x)

[62] Gasparini Z, De la Fuente MS 2000 Tortugas y plesiosaurios de la Formación La Colonia (Cretácico Superior) de Patagonia. Rev. Española Paleontol. 15, 23–35.

[63] Benson RBJ, Evans M, Druckenmiller PS 2012 High diversity, low disparity and small body size in plesiosaurs (Reptilia, Sauropterygia) from the Triassic–Jurassic boundary. PLoS ONE 7, e31838 (doi:10.1371/journal.pone.0031838) 22438869 10.1371/journal.pone.0031838 PMC3306369

[64] Sato T, Storrs GW 2000 An early polycotylid plesiosaur (Reptilia: Sauropterygia) from the Cretaceous of Hokkaido, Japan. J. Paleontol. 74, 907–914. (doi:10.1017/S0022336000033096)

[65] Vincent P, Weis R, Kronz G, Delsatte D 2017 *Microcleidus melusinae*, a new plesiosaurian (Reptilia, Plesiosauria) from the Toarcian of Luxembourg. Geol. Mag. (online) (doi:10.1017/S0016756817000814)

[66] Noè LF, Taylor MA, Gómez-Pérez M 2017 An integrated approach to understanding the role of the long neck in plesiosaurs. Acta Palaeontol. Pol. 62, 137–162. (doi:10.4202/app.00334.2016)

[67] Kear BP 2016 Cretaceous marine amniotes of Australia: perspectives on a decade of new research. Mem. Museum Victoria Pap. Palaeontol. 74, 17–28. (doi:10.24199/j.mmv.2016.74.03)

[68] Benson RBJ, Bowdler T 2014 Anatomy of *Colymbosaurus megadeirus* (Reptilia, Plesiosauria) from the Kimmeridge Clay Formation of the U.K., and high diversity among Late Jurassic plesiosauroids. J. Vertebr. Paleontol. 34, 1053–1071. (doi:10.1080/02724634.2014.850087)

[69] Gasparini Z, Sterli J, Parras A, O'Gorman JP, Salgado L, Varela J, Pol D 2015 Late Cretaceous reptilian biota of the La Colonia Formation, central Patagonia, Argentina: Occurrences, preservation and paleoenvironments. Cretac. Res. 54, 154–168. (doi:10.1016/j.cretres.2014.11.010)

[70] Fischer V, Bardet N, Benson RBJ, Arkhangelsky MS, Friedman M 2016 Extinction of fish-shaped marine reptiles associated with reduced evolutionary rates and global environmental volatility. Nat. Commun. 7, 1–11. (doi:10.1038/ncomms10825) 10.1038/ncomms10825 PMC4786747 26953824

[71] Andrews CW 1909 On some new Plesiosauria from the Oxford Clay of Peterborough. Ann. Mag. Nat. Hist. Zool. Bot. Geol. 4, 418–429. (doi:10.1080/00222930908692691)

[72] Sollas WJ 1881 On a new species of *Plesiosaurus* (*P. conybeari*) from the Lower Lias of Charmouth; with observations on *P. megacephalus*, *Stutchbury*, and *P. brachycephalus*, Owen. Q. J. Geol. Soc. 37, 440–481. (doi:10.1144/GSL.JGS.1881.037.01-04.42)

[73] Adams DA 1997 *Trinacromerum bonneri*, new species, last and fastest pliosaur of the Western Interior Seaway. Texas J. Sci. 49, 179–198.

[74] Bakker RT 1993 Plesiosaur extinction cycles — events that mark the Beginning, Middle and End of the Cretaceous. In Evolution of the western interior basin: Geological Association of Canada, special paper (eds Caldwell WGE, Kauffman EG), pp. 641–664. Stittsville, Ontario, Canada.

[75] Bardet N 1992 Stratigraphic evidence for the extinction of the ichthyosaurs. Terra Nov. 4, 649–656. (doi:10.1111/j.1365-3121.1992.tb00614.x)

[76] Schumacher BA 2011 A ‘woollgari-zone mosasaur’ (Squamata; Mosasauridae) from the Carlile Shale (Lower Middle Turonian) of central Kansas and the stratigraphic overlap of early mosasaurs and pliosaurid plesiosaurs. Trans. Kansas Acad. Sci. 114, 1–14. (doi:10.1660/062.114.0101)

[77] Kear BP 2003 Cretaceous marine reptiles of Australia: a review of taxonomy and distribution. Cretac. Res. 24, 277–303. (doi:10.1016/S0195-6671(03)00046-6)

[78] Kear BP 2004 Biogeographic and biostratigraphic implications of Australian Mesozoic marine reptiles. Aust. Biol. 17, 4–22.

[79] Fischer V 2016 Taxonomy of *Platypterygius campylodon* and the diversity of the last ichthyosaurs. PeerJ 4, 1–21. (doi:10.7717/peerj.2604) 10.7717/peerj.2604 PMC5075704 27781178

[80] Prokoph A, Shields GA, Veizer J 2008 Compilation and time-series analysis of a marine carbonate *δ*^18^O, *δ*^13^C, ^87^Sr/^86^Sr and *δ*^34^S database through Earth history. Earth Sci. Rev. 87, 113–133. (doi:10.1016/j.earscirev.2007.12.003)

[81] Conybeare WD 1824 On the discovery of an almost perfect skeleton of the *Plesiosaurus*. Trans. Geol. Soc. Lond. Second Ser. 1, 381–389. (doi:10.1144/transgslb.1.2.381)

[82] Massare JA 1988 Swimming capabilities of Mesozoic marine reptiles: implications for the methods of predation. Palaeobiology 14, 187–205. (doi:10.1017/S009483730001191X)

[83] Bardet N, Houssaye A, Rage J-C, Suberbiola XP 2008 The Cenomanian-Turonian (Late Cretaceous) radiation of marine squamates (Reptilia): the role of the Mediterranean Tethys. Bull. Soc. Géol. Fr. 179, 605–622. (doi:10.2113/gssgfbull.179.6.605)

[84] Polcyn MJ, Jacobs LL, Araújo R, Schulp AS, Mateus O 2014 Physical drivers of mosasaur evolution. Palaeogeogr. Palaeoclimatol. Palaeoecol. 400, 17–27. (doi:10.1016/j.palaeo.2013.05.018)

[85] Monnet C 2009 The Cenomanian-Turonian boundary mass extinction (Late Cretaceous): new insights from ammonoid biodiversity patterns of Europe, Tunisia and the Western Interior (North America). Palaeogeogr. Palaeoclimatol. Palaeoecol. 282, 88–104. (doi:10.1016/j.palaeo.2009.08.014)

[86] Monnet C, Bucher H 2007 European ammonoid diversity questions the spreading of anoxia as primary cause for the Cenomanian/Turonian (Late Cretaceous) mass extinction. Swiss J. Geosci. 100, 137–144. (doi:10.1007/s00015-007-1209-1)

[87] Kurihara K, Toshimitsu S, Hirano H 2012 Ammonoid biodiversity changes across the Cenomanian/Turonian boundary in the Yezo Group, Hokkaido, Japan. Acta Palaeontol. Pol. 57, 749–757. (doi:10.4202/app.2011.0064)

[88] Skelton PW 2003 Rudist evolution and extinction — a North African perspective. In North African Cretaceous carbonate platform systems: proceedings of the NATO advanced research workshop, Tunis, Tunisia 13–18 May 2002 (eds Gili E, Negra MEH, Skelton PW), pp. 215–227. Dordrecht, the Netherlands: Kluwer Academic Publishers.

[89] Skelton PW, Spicer RA, Kelley S, Gilmour I 2003 The Cretaceous world. New York, NY: Cambridge University Press.

[90] Bilotte M 1989 Comparaisons entre événements sédimentologiques et biologiques dans le Crétacé moyen (Vraconien-Turonien) des plates-formes est-pyrénéennes. In Les événements de la partie moyenne du Crétacé (Aptien à Turonien) (ed. Cotillon P), pp. 255–266. Lyon, France: Edition de l'Université Claude-Bernard.

[91] Cavin L, Forey PL 2007 Using ghost lineages to identify diversification events in the fossil record. Biol. Lett. 3, 201–204. (doi:10.1098/rsbl.2006.0602) 17284405 10.1098/rsbl.2006.0602 PMC2375936

[92] Cavin L, Forey PL, Lécuyer C 2007 Correlation between environment and Late Mesozoic ray-finned fish evolution. Palaeogeogr. Palaeoclimatol. Palaeoecol. 245, 353–367. (doi:10.1016/j.palaeo.2006.08.010)

[93] Bambach RK 2006 Phanerozoic biodiversity mass extinctions. Annu. Rev. Earth Planet. Sci. 34, 127–155. (doi:10.1146/annurev.earth.33.092203.122654)

[94] Guinot G, Adnet S, Cappetta H 2012 An analytical approach for estimating fossil record and diversification events in sharks, skates and rays. PLoS ONE 7, e44632 (doi:10.1371/journal.pone.0044632) 22957091 10.1371/journal.pone.0044632 PMC3434181

[95] Guinot G 2013 Regional to global patterns in Late Cretaceous selachian (Chondrichthyes, Euselachii) diversity. J. Vertebr. Paleontol. 33, 521–531. (doi:10.1080/02724634.2013.740116)

[96] Fischer V, Benson RBJ, Druckenmiller PS, Ketchum HF, Bardet N 2018 Data from: The evolutionary history of polycotylid plesiosaurians. Dryad Digital Repository (http://dx.doi.org/10.5061/dryad.4gh8b) 10.1098/rsos.172177 PMC5882735 29657811

