# Supplementary material for: The evolutionary history of polycotylid plesiosaurians
Source: R Soc Open Sci. 2018 Mar 28;5(3):172177. doi: 10.1098/rsos.172177 (PMC5882735; doi:10.1098/rsos.172177)
Supplement: Supplementary Information [file rsos172177supp1.docx]

**The evolutionary history of polycotylid plesiosaurians**

**Fischer V.^1,^*, Benson R.B.J.^2,^*, Druckenmiller P.S.^3^, Ketchum H.F.^4^ & Bardet N^5^.**

**SUPPLEMENTARY INFORMATION**

**SUPPLEMENTARY METHODS: BAUPLAN DATA**

We analysed the following taxa (Table S1):

**Table S1**. Source data for the Bauplan dataset and relative completeness of each taxon. The taxa written in blue passed the 50% completeness threshold while taxa in red did not and were not used in the subsequent analyses. Data was collected on *Manemergus anguirostris* and is presented here, but this taxon was not used in our analyses of morphological disparity and morphospace occupation because of its juvenile status, which can artificially inflate the disparity and morphological range by showing a juvenile, plesiomorphic-like morphology among Turonian polycotylids. Indeed, while this issue has yet to investigated in detail, highly juvenile marine reptiles tend to preserve a mosaic of plesiomorphic and more derived traits (e.g. [1,2]).

| Taxon | Data sources | Completeness |
| --- | --- | --- |
| *Edgarosaurus muddi* | Own measurements on photographs of MOR 751 (holotype); [3] | 77% |
| *Plesiopleurodon wellesi* | Own measurements on fossil and on photographs of CM 2815 (holotype); [4,5] | 66% |
| ‘Richmond pliosaur’ | Own measurements on fossil and on photographs of QM F1609; [5] | 100% |
| *Palmulasaurus quadratus* | Own measurements on photographs of MNA V9442; [6]; Humerus/femur from [7] | 11% |
| *Pahasapasaurus haasi* | [8] (specimen MNA V9442 [holotype]) | 66% |
| *Polycotylus latipinnis* | [9] (for cranium on specimen SDSM 23020) and [10] (for neck/skull ratio on specimen YPM 1125) | 100% |
| *Thililua longicollis* | Own measurements on fossil of MHNGr.PA. (holotype); [11] | 66% |
| *Trinacromerum bentonianum* | [8] (mean of specimens KUVP5070 and YPM1129); [10] (for neck/skull ratio); [12] (for symphysial teeth) | 66% |
| *Dolichorhynchops osborni* | [8] (mean of several specimens); [10]; own measurements on photographs of KUVP1300 (holotype) for propodials and neck/skull; [12] (for symphysial teeth) | 88% |
| *Dolichorhynchops bonneri* | [13] (paratype specimen KUVP 40001): Adams 1997; own measurements on photographs of KUVP 40001; [12] (for symphysial teeth) | 77% |
| *Eopolycotylus rankini* | Own measurements on photographs of MNA V9445; [6] | 22% |
| *Dolichorhynchops tropicensis* | [7] (specimen MNA V10046) | 88% |
| *Dolichorhynchops herschelensis* | [14] (specimen RSM P2310.1; holotype) | 44% |
| *Sulcusuchus erraini* | [15] (specimen MPEF 650) | 0% |
| *Mauriciosaurus fernandezi* | [16] (specimen CPC RFG 2544 P.F.1) | 66% |
| *Manemergus anguirostris* | Own measurements on fossil and on photographs of SMNK 3861 (holotype); [17] | 88% |

We took the following twelve measurements using, when possible, a calliper. Measurements above 100 mm have been taken using a meter tape and using the software ImageJ [18] on high-quality undistorted photographs (Table S2):

1. Mandible anteroposterior length
2. Skull mediolateral width (combining both lateral sides) anterior to the orbit
3. Snout anteroposterior length anterior to the orbit
4. Symphysis anteroposterior length
5. Number of symphysial teeth
6. Tooth apicobasal height (crown + root)
7. Crown apicobasal height
8. Crown maximal diameter
9. Neck anteroposterior length
10. Trunk anteroposterior length
11. Humerus proximodistal length
12. Femur proximodistal length

**Table S2**. Measurements used for the Bauplan dataset (see Table S3). All measurements are in mm. The taxa written in blue passed the 50% completeness threshold while taxa in red did not and were not used in subsequent analyses. See Table S1 above for explanations on the treatment of *Manemergus anguirostris*.

| **Taxon** | **Mandible length** | **Skull width anterior to orbit** | **Snout length** | **Symphysis length** | **Symph teeth** | **Tooth size** | **Crown height** | **Crown width** | **Neck length** | **Trunk length** | **Humerus length** | **Femur length** |
| --- | --- | --- | --- | --- | --- | --- | --- | --- | --- | --- | --- | --- |
| *Edgarosaurus muddi* | 520 | 80 | 255.075 | 145 | 6 | NA | 49 | 15 | 704 | NA | NA | NA |
| *Plesiopleurodon wellesi* | 784 | 152 | 313 | 195 | 8 | NA | 41.4 | 18 | NA | NA | NA | NA |
| *Richmond pliosaur* | 845 | 171 | 425 | 284 | 12 | NA | 21.7 | 11.7 | 1380 | 1330 | 400 | 407 |
| *Palmulasaurus quadratus* | NA | NA | NA | NA | 10 | NA | NA | NA | NA | NA | 296 | 270 |
| *Pahasapasaurus haasi* | 900 | 173 | 625 | 380 | 12 | NA | NA | NA | NA | NA | 450 | 490 |
| *Polycotylus latipinnis* | 1000 | 185 | 635 | 450 | 18 | 77 | 24 | 12 | 1120 | NA | 488 | 503 |
| *Thililua longicollis* | 665 | NA | 392.5 | 290 | 15 | 41 | 31 | 11.5 | 2167 | NA | NA | NA |
| *Trinacromerum bentonianum* | 708.5 | NA |  | 293.5 | 11 | NA | 22.9 | 10.14 | 1070 | NA | NA | NA |
| *Dolichorhynchops osborni* | 710.25 | NA | 495.5 | 321.25 | 18.5 | NA | 23.8 | 9 | 710 | NA | 210 | 275 |
| *Dolichorhynchops bonneri* | 983 | NA | 619 | 440 | 19 | 80.88 | 24.24 | 14.16 | NA | 1480 | 578 | 520 |
| *Eopolycotylus rankini* | NA | NA | NA | 284 | 14.5 | NA | NA | NA | NA | NA | 250 | 380 |
| *Dolichorhynchops tropicensis* | 627 | 95.1 | 346 | 265 | NA | 56.4 | 24.18 | 7.6 | 685 | NA | 255 | 260 |
| *Dolichorhynchops herschelensis* | 510 | 113.2 | 265.44 | 225.3 | 19 | NA | NA | NA | NA | NA | NA | NA |
| *Sulcusuchus erraini* | NA | NA | NA | NA | NA | NA | NA | NA | NA | NA | NA | NA |
| *Mauriciosaurus fernandezi* | 422.5 | NA | NA | 175 | NA | NA | 12 | 3 | 391 | 644 | 175 | 182.5 |
| *Manemergus anguirostris* | 315 | 40 | 155.04 | 130 | 9 | NA | 12 | 5 | 610 | 700 | NA | 135 |

We used this data to produce a series of ratios, which we combine with one absolute measurement (crown apicobasal height); all these characters (nine in total) are ecologically relevant (Table S3):

- Relative snout width (rostrum width anterior to the orbit divided by mandible anteroposterior length)
- Relative snout length (rostrum anteroposterior length anterior to the orbit divided by mandible length)
- Relative symphysis length (symphysis length divided by mandible length)
- Density of symphysial teeth (number of symphysial teeth divided by the symphysis length); an easy to compute proxy for tooth density
- Absolute crown height of the largest tooth (a crucial determinant in the diet of odontocete cetaceans [e.g. ,19])
- Crown shape (crown apicobasal height divided by the basal diameter of the crown, largest tooth)
- Homogeneity of flipper sizes (humerus proximodistal length divided by femur proximodistal length)
- Flipper size (mandible anteroposterior length divided by femur length)
- Neck/skull length ratio (neck length divided by mandible anteroposterior length)

**Table S3**. Bauplan dataset. All characters are ratios, except “CROWN HEIGHT” which is an absolute value. See ESM7 ecodata.txt for a ready-to-use text file.

|  | Snout width | Relative snout | Relative symphysis | Symphial teeth density | Crown height | Crown shape | Humerus/femur | Mandible/femur |
| --- | --- | --- | --- | --- | --- | --- | --- | --- |
| *Edgarosaurus muddi* | 0.153846154 | 0.490528846 | 0.278846154 | 0.04137931 | 49 | 3.266666667 | NA | NA |
| *Plesiopleurodon wellesi* | 0.193877551 | 0.399234694 | 0.24872449 | 0.041025641 | 41.4 | 2.3 | NA | NA |
| Richmond pliosaur | 0.202366864 | 0.50295858 | 0.336094675 | 0.042253521 | 21.7 | 1.854700855 | 0.982800983 | 2.076167076 |
| *Palmulasaurus quadratus* | NA | NA | NA | NA | NA | NA | 1.096296296 | NA |
| *Pahasapasaurus haasi* | 0.192222222 | 0.694444444 | 0.422222222 | 0.031578947 | NA | NA | 0.918367347 | 1.836734694 |
| *Polycotylus latipinnis* | 0.185 | 0.635 | 0.45 | 0.04 | 24 | 2 | 0.970178926 | 1.988071571 |
| *Thililua longicollis* | NA | 0.590225564 | 0.436090226 | 0.051724138 | 31 | 2.695652174 | NA | NA |
| *Trinacromerum bentonianum* | NA | 0.588 | 0.414255469 | 0.037478705 | 22.9 | 2.258382643 | NA | NA |
| *Dolichorhynchops osborni* | NA | 0.697641675 | 0.452305526 | 0.057587549 | 23.8 | 2.644444444 | 0.763636364 | 2.582727273 |
| *Dolichorhynchops bonneri* | NA | 0.629704985 | 0.447609359 | 0.043181818 | 24.24 | 1.711864407 | 1.111538462 | 1.890384615 |
| *Eopolycotylus rankini* | NA | NA | NA | 0.051056338 | NA | NA | 0.657894737 | NA |
| *Dolichorhynchops tropicensis* | 0.151674641 | 0.551834131 | 0.422647528 | NA | 24.18 | 3.181578947 | 0.980769231 | 2.411538462 |
| *Dolichorhynchops herschelensis* | 0.221960784 | 0.520470588 | 0.441764706 | 0.084332002 | NA | NA | NA | NA |
| *Sulcusuchus erraini* | NA | NA | NA | NA | NA | NA | NA | NA |
| *Mauriciosaurus fernandezi* | NA | NA | 0.414201183 | NA | 12 | 4 | 0.95890411 | 2.315068493 |

**SUPPLEMENTARY RESULTS AND FIGURES**

**Phylogenetic analyses**

**Table S4**. Results of the phylogenetic diversity estimates. We have computed the median phylogenetic diversity estimate and the 95% confidence interval using both an ‘equal’ (“eq”)


|  | Full_eq_median | Full_eq_05 | Full_eq_95 | Full_Ba_median | Full_Ba_05 | Full_Ba_95 | Red_eq_median | Red_eq_05 | Red_eq_95 | Red_Ba_median | Red_Ba_05 | Red_Ba_95 |
| --- | --- | --- | --- | --- | --- | --- | --- | --- | --- | --- | --- | --- |
| Maa | 2 | 2 | 2 | 2 | 2 | 2 | 2 | 2 | 2 | 2 | 2 | 2 |
| Cam | 5 | 5 | 5 | 5 | 5 | 5 | 5 | 5 | 5 | 5 | 5 | 5 |
| San | 7 | 7 | 7 | 7 | 7 | 7 | 6 | 6 | 6 | 5.5 | 5 | 6 |
| Con | 7 | 7 | 7 | 3 | 3 | 3 | 6 | 5 | 6 | 4 | 3 | 4 |
| Tur | 12 | 12 | 12 | 10 | 10 | 10 | 11.5 | 11 | 12 | 10 | 9 | 10 |
| Cen | 12 | 12 | 12 | 9 | 8 | 9 | 12.5 | 12 | 13 | 11 | 10 | 11 |
| Upper_Alb | 11 | 11 | 11 | 8 | 7 | 9 | 13 | 12 | 13 | 7 | 6 | 7 |
| Mid_Alb | 11 | 10 | 11 | 1 | 1 | 1 | 7 | 6 | 7 | 1 | 1 | 1 |
| Lower_Alb | 10 | 9 | 11 | 1 | 1 | 1 | 7 | 6 | 7 | 1 | 1 | 1 |
| U_pperApt | 9 | 7 | 9 | 1 | 1 | 1 | 6 | 5 | 6 | 1 | 1 | 1 |
| Lower_Apt | 5 | 4 | 6 | 1 | 1 | 1 | 4 | 4 | 4 | 1 | 1 | 1 |
| Bar | 4 | 4 | 5 | 1 | 1 | 1 | 4 | 3 | 4 | 1 | 1 | 1 |
| Hau | 3 | 3 | 3 | 1 | 1 | 1 | 3 | 2 | 3 | 1 | 1 | 1 |
| Val | 2 | 2 | 2 | 1 | 1 | 1 | 2 | 2 | 2 | 1 | 1 | 1 |
| Ber | 1 | 1 | 1 | 0 | 0 | 0 | 1 | 1 | 1 | 0 | 0 | 0 |

**Figure S1.** Phylogeny of plesiosaurians. Strict consensus of the 50000 most parsimonious trees resulting from the maximum parsimony analysis of the full dataset. Timescaled and generated using the ape v5.0 [20], paleotree v3.0 [21], phangorn v2.31 [22], strap v1.4 [23], Claddis v0.2 [24] packages.

**Figure S2.** Stratigraphic congruence index (Gap Excess Ratio) for the 50000 most parsimonious trees arising from the maximum parsimony analysis of the full dataset, compared to a set 1000 randomly generated trees using the same biostratigraphic data. Computed using the strap v1.4 [23] package.

**Figure S3.** Phylogeny of plesiosaurians. Strict consensus of the 3584 most parsimonious trees resulting from the maximum parsimony analysis of the pruned dataset. Timescaled and generated using the ape v5.0 [20], paleotree v3.0 [21], phangorn v2.31 [22], strap v1.4 [23], Claddis v0.2 [24] packages.

**Figure S4.** Stratigraphic congruence index (Gap Excess Ratio) for the 3584 most parsimonious trees arising from the maximum parsimony analysis of the pruned dataset, compared to a set 1000 randomly generated trees using the same biostratigraphic data. Computed using the strap v1.4 [23] package.

**Morphospace**

**Figure S5.** Phylomorphospace of polycotylids (using the phylogenetic tree arising the analysis of the pruned dataset with the best Gap Excess Ratio score and a pruned Bauplan dataset that contain the species present in both the Bauplan dataset and the phylogeny). Computed using the phytools v0.6-44 [25], paleotree v3.0 [21] and zoo v1.8 [26] packages.

**Table S5**. Evolution over time of the disparity of Polycotylidae (sum of variances) and 95% confidence interval using Bauplan data. The bins used are Aptian, Albian, Cenomanian, Turonian, Coniacian-Santonian, Campanian-Maastrichtian.

|  | Sum of variance | Lower bound CI | Upper bound CI |
| --- | --- | --- | --- |
| Campanian-Maastrichtian | 9.219733806 | 4.461016545 | 14.77631106 |
| Coniacian-Santonian | NA | NA | NA |
| Turonian | 9.267559693 | 4.530608382 | 15.02734417 |
| Cenomanian | 12.8032765 | 9.54064645 | 20.34934552 |
| Albian | 10.96863605 | 4.091184171 | 19.06406869 |
| Aptian | NA | NA | NA |

**REFERENCES**

1. Kear BP, Zammit M. 2014 In utero foetal remains of the Cretaceous ichthyosaurian *Platypterygius*: ontogenetic implications for character state efficacy. *Geol. Mag.* **151**, 71–86. (doi:10.1017/S0016756813000113)

2. Maxwell EE, Caldwell MW. 2006 A new genus of ichthyosaur from the Lower Cretaceous of Western Canada. *Palaeontology* **49**, 1043–1052.

3. Druckenmiller PS. 2002 Osteology of a new plesiosaur from the Lower Cretaceous (Albian) Thermopolis Shale of Montana. *J. Vertebr. Paleontol.* **22**, 29–42.

4. Carpenter K. 1996 A review of short-necked plesiosaurs from the Cretaceous of the Western Interior, North America. *Neues Jahrb. für Geol. und Paläontologie, Abhandlungen* **201**, 259–287.

5. Fischer V, Benson RBJ, Zverkov NG, Soul LC, Arkhangelsky MS, Lambert O, Stenshin IM, Uspensky GN, Druckenmiller PS. 2017 Plasticity and convergence in the evolution of short-necked plesiosaurs. *Curr. Biol.* **27**, 1667–1676. (doi:10.1016/j.cub.2017.04.052)

6. Albright LB, Gillette DD, Titus AL. 2007 Plesiosaurs from the Upper Cretaceous (Cenomanian–Turonian) Tropic Shale of southern Utah, part 2: Polycotylidae. *J. Vertebr. Paleontol.* **27**, 41–58. (doi:10.1671/0272-4634(2007)27[41:PFTUCC]2.0.CO;2)

7. McKean R. 2012 A new species of polycotylid plesiosaur (Reptilia: Sauropterygia) from the Lower Turonian of Utah: Extending the stratigraphic range of *Dolichorhynchops*. *Cretac. Res.* **34**, 184–199.

8. Schumacher BA. 2007 A new polycotylid plesiosaur (Reptilia; Sauropterygia) from the Greenhorn Limestone (Upper Cretaceous; lower upper Cenomanian), Black Hills, South Dakota. *Geol. Soc. Am. Spec. Pap.* **427**, 133–146.

9. Schumacher BA, Martin JE. 2015 *Polycotylus latipinnis* Cope (Plesiosauria, Polycotylidae), a nearly complete skeleton from the Niobrara Formation (Early Campanian) of southwestern South Dakota. *J. Vertebr. Paleontol.* , e1031341. (doi:10.1080/02724634.2015.1031341)

10. O’Keefe FR. 2002 The evolution of plesiosaur and pliosaur morphotypes in the Plesiosauria (Reptilia: Sauropterygia). *Palaeobiology* **28**, 101–112.

11. Bardet N, Pereda Suberbiola X, Jalil N-E. 2003 A new polycotylid plesiosaur from the Late Cretaceous (Turonian) of Morocco. *Comptes rendus Palevol* **2**, 307–315.

12. O’Keefe RF. 2008 Cranial anatomy and taxonomy of *Dolichorhynchops bonneri* new combination, a poltycotylid (Sauropterygia: Plesiosauria) from the Pierre Shale of Wyoming and South Dakota. *J. Vertebr. Paleontol.* **28**, 664–676.

13. Adams DA. 1997 *Trinacromerum bonneri*, new species, last and fastest pliosaur of the Western Interior Seaway. *Texas J. Sci.* **49**, 179–198.

14. Sato T. 2005 A new polycotylid plesiosaur (Reptilia: Sauropterygia) from the Upper Cretaceous Bearpaw Formation in Saskatchewan, Canada. *J. Paleontol.* **79**, 969–980.

15. O’Gorman JP, Gasparini Z. 2013 Revision of *Sulcusuchus erraini* (Sauropterygia, Polycotylidae) from the Upper Cretaceous of Patagonia, Argentina. *Alcheringa An Australas. J. Palaeontol.* **37**, 163–176. (doi:10.1080/03115518.2013.736788)

16. Frey E, Mulder EWA, Stinnesbeck W, Rivera-sylva HE, Padilla-gutiérrez JM, González-gonzález AH. 2017 A new polycotylid plesiosaur with extensive soft tissue preservation from the early Late Cretaceous of northeast Mexico. *Boletín la Soc. Geológica Mex.* **69**, 87–134.

17. Buchy M-C, Métayer F, Frey E. 2005 Osteology of *Manemergus anguirostris* n.gen. et sp., a new plesiosaur (Reptilia, Sauropterygia) from the Upper Cretaceous of Morocco. *Palaeontographica* **272**, 97–120.

18. Abràmoff MD, Magalhães PJ, Ram SJ. 2004 Image processing with imageJ. *Biophotonics Int.* **11**, 36–41.

19. Ridgway SH, Harrison R. 1999 *The Second Book of Dolphins and the Porpoise*. San Diego: Academic Press.

20. Paradis E, Claude J, Strimmer K. 2004 APE: Analyses of phylogenetics and evolution in R language. *Bioinformatics* **20**, 289–290.

21. Bapst DW. 2012 paleotree: an R package for paleontological and phylogenetic analyses of evolution. *Methods Ecol. Evol.* **3**, 803–807. (doi:10.1111/j.2041-210X.2012.00223.x)

22. Schliep KP. 2011 phangorn: phylogenetic analysis in R. *Bioinformatics* **27**, 592–593. (doi:10.1093/bioinformatics/btq706)

23. Bell MA, Lloyd GT. 2015 strap : an R package for plotting phylogenies against stratigraphy and assessing their stratigraphic congruence. *Palaeontology* **58**, 379–389. (doi:10.1111/pala.12142)

24. Lloyd GT. 2015 Package ‘Claddis’. , 1–24.

25. Revell LJ. 2012 phytools: An R package for phylogenetic comparative biology (and other things). *Methods Ecol. Evol.* **E**, 217–223.

26. Zeileis A, Grothendieck G. 2005 zoo: S3 Infrastructure for Regular and Irregular Time Series. *J. Stat. Softw.* **14**, 1–27.
